# The Steroid Catabolic Pathway of the Intracellular Pathogen *Rhodococcus equi* Is Important for Pathogenesis and a Target for Vaccine Development

**DOI:** 10.1371/journal.ppat.1002181

**Published:** 2011-08-25

**Authors:** R. van der Geize, A. W. F. Grommen, G. I. Hessels, A. A. C. Jacobs, L. Dijkhuizen

**Affiliations:** 1 Groningen Biomolecular Sciences and Biotechnology Institute (GBB), Department of Microbiology, University of Groningen, Groningen, The Netherlands; 2 Intervet International BV, Microbiological R&D, Boxmeer, The Netherlands; Harvard School of Public Health, United States of America

## Abstract

*Rhodococcus equi* causes fatal pyogranulomatous pneumonia in foals and immunocompromised animals and humans. Despite its importance, there is currently no effective vaccine against the disease. The actinobacteria *R. equi* and the human pathogen *Mycobacterium tuberculosis* are related, and both cause pulmonary diseases. Recently, we have shown that essential steps in the cholesterol catabolic pathway are involved in the pathogenicity of *M. tuberculosis*. Bioinformatic analysis revealed the presence of a similar cholesterol catabolic gene cluster in *R. equi*. Orthologs of predicted *M. tuberculosis* virulence genes located within this cluster, i.e. *ipdA* (*rv3551), ipdB* (*rv3552)*, *fadA6* and *fadE30*, were identified in *R. equi* RE1 and inactivated. The *ipdA* and *ipdB* genes of *R. equi* RE1 appear to constitute the α-subunit and β-subunit, respectively, of a heterodimeric coenzyme A transferase. Mutant strains RE1Δ*ipdAB* and RE1Δ*fadE30*, but not RE1Δ*fadA6*, were impaired in growth on the steroid catabolic pathway intermediates 4-androstene-3,17-dione (AD) and 3aα-H-4α(3′-propionic acid)-5α-hydroxy-7aβ-methylhexahydro-1-indanone (5α-hydroxy-methylhexahydro-1-indanone propionate; 5OH-HIP). Interestingly, RE1Δ*ipdAB* and RE1Δ*fadE30*, but not RE1Δ*fadA6*, also displayed an attenuated phenotype in a macrophage infection assay. Gene products important for growth on 5OH-HIP, as part of the steroid catabolic pathway, thus appear to act as factors involved in the pathogenicity of *R. equi.* Challenge experiments showed that RE1Δ*ipdAB* could be safely administered intratracheally to 2 to 5 week-old foals and oral immunization of foals even elicited a substantial protective immunity against a virulent *R. equi* strain. Our data show that genes involved in steroid catabolism are promising targets for the development of a live-attenuated vaccine against *R. equi* infections.

## Author Summary

*Rhodococcus equi* causes fatal pyogranulomatous bronchopneumonia in young foals and is an emerging opportunistic pathogen of immunocompromised humans. Despite its importance, there is currently no safe and effective vaccine against *R. equi* infections. Like *Mycobacterium tuberculosis*, the causative agent of human tuberculosis, *R. equi* is able to infect, survive and multiply inside alveolar macrophages. Recently we have shown that essential steps in the cholesterol catabolic pathway (encoded by the *rv3551*, *rv3552*, *fadE30* genes) are involved in the pathogenicity of *M. tuberculosis*. We hypothesized that the orthologous genes in the cholesterol catabolic gene cluster of *R. equi* also are essential for its virulence mechanism. Analysis of the respective *R. equi* strain RE1 mutants revealed that they were impaired in growth on intermediates of the steroid catabolic pathway and had attenuated phenotypes in a macrophage infection assay. Mutant RE1Δ*ipdAB*, carrying a deletion of the orthologs of *rv3551* and *rv3552*, could be safely administered to 2–5 week-old foals intratracheally and oral immunization provided a substantial protection against infection by a virulent *R. equi* strain. Our data show that genes important for methylhexahydroindanone propionate degradation, part of the steroid catabolic pathway, are promising targets for the development of a live-attenuated vaccine against *R. equi* infections.

## Introduction

*Rhodococcus equi* is a nocardioform Gram-positive bacterium and a facultative intracellular pathogen that causes fatal pyogranulomatous bronchopneumonia in young foals aged up to five months. *R. equi* is also an emerging opportunistic pathogen of immunocompromised humans, particularly HIV infected patients [1]–[3]. Like *Mycobacterium tuberculosis*, the causative agent of tuberculosis (TB) in man, *R. equi* is able to infect, survive and multiply inside the host cells mainly in alveolar macrophages [4]–[7]. *R. equi* and *M. tuberculosis* are both members of the class of *Actinomycetales* and share physical, biochemical and cell biological characteristics [2]. Antibiotic treatment of *R. equi* infections is not consistently successful and is costly due to the necessity of treatment for a prolonged period of time [8]. More importantly, there is currently no safe and effective vaccine against *R. equi* infections.

Virulence of *R. equi* is dependent on the presence of a plasmid (approx. 85–95 kb) which is essential for *R. equi* to survive and grow in macrophages [9]–[13]. This virulence plasmid carries a pathogenicity island, encoding a number of related virulence associated proteins (Vaps) that includes the immunodominant surface expressed protein VapA [9], [10], [14]. Following infection with *R. equi*, the presence of the VapA-expressing virulence-associated plasmid is believed to promote necrotic damage to the host, which is strongly pro-inflammatory [15], [16]. VapA is not required for host cell necrosis, but has been implicated in early phagosome development [17]. Consistent with this role, mutational analysis showed that *vapA*, unlike *vapG*, is indispensable for multiplication of *R. equi* in macrophages and its persistence in mice [12], [18]. Indeed, VapA has been most widely investigated in vaccine studies for the prevention of *R. equi* infections. Oral vaccination of mice with an attenuated *Salmonella enterica* Typhimurium vaccine strain expressing VapA protein, for example, has been shown to confer protection against virulent *R. equi* [19], [20]. DNA vaccines encoding *vapA* have also been shown to stimulate cell-mediated immunity [21], [22].

Besides the *vap* genes, only a limited number of other virulence genes have been identified in *R. equi* to date. Random transposon mutagenesis using *Himar1* transposition in *R. equi* identified a metabolic gene essential for riboflavin biosynthesis. The riboflavin auxotrophic mutant was shown to be fully attenuated in immunocompromised mice and could be safely administered to young foals [23], [24]. Immunization of young foals with the riboflavin auxotrophic mutant, however, did not afford protection against a virulent *R. equi* challenge [24]. *choE*, encoding the extracellular cholesterol oxidase in *R. equi*, is believed to be involved in macrophage destruction [25], but is not essential for virulence [26], [27]. Isocitrate lyase, a key enzyme of the glyoxylate bypass encoded by *aceA*, was shown to be important for virulence of *R. equi*. An *aceA* mutant was unable to proliferate in macrophages, was attenuated in mice and, when administrated to 3-week-old foals, did not induce pneumonic disease [28]. Crucially, a *choE aceA* double mutant in some cases was still able to induce severe pneumonia in 1-week-old foals, indicating that the mutant was not fully safe [27]. Attenuated mutants of *R. equi* were also obtained by targeted mutagenesis of *htrA, narG*, or *pepD* [29]. *pepD* in *M. tuberculosis* H37Rv is controlled by the two-component regulatory system MprA-MprB [30]. Consistent with this, the sensor kinase MprB of *R. equi* 103 was recently found to be required for intracellular survival [31]. So far, however, none of the strategies or identified virulence factors has resulted in the development of a safe vaccine capable of providing protective immunity against *R. equi* infection in young foals.

**Figure 1:**
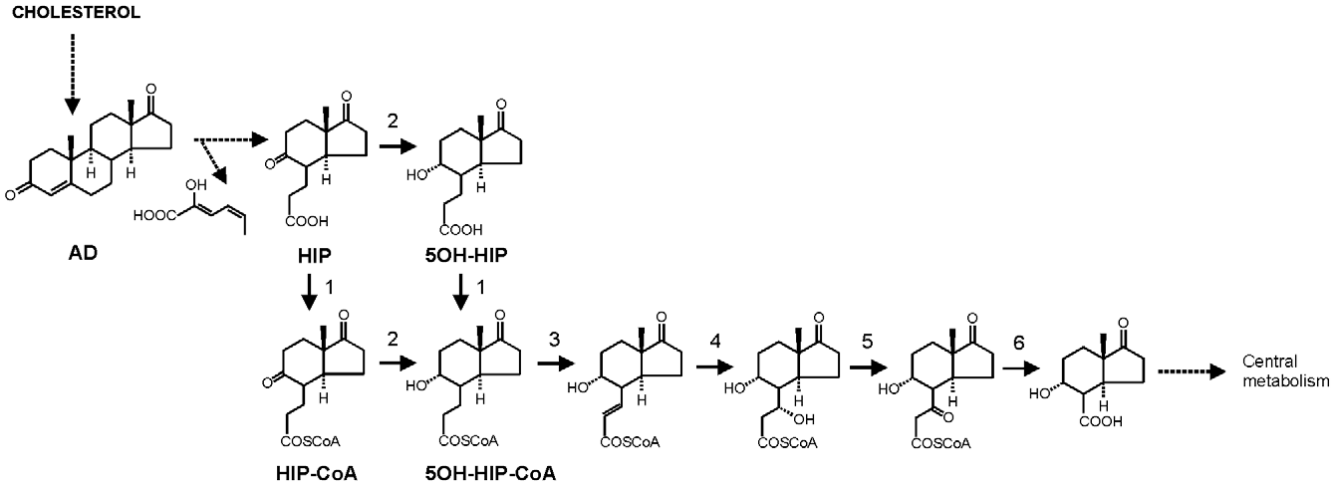
Proposed pathway of 4-androstene-3,17-dione (AD) degradation via β-oxidation of methylhexahydroindanone propionate intermediates 3aα-H-4α(3′-propionic acid)-7aβ-methylhexahydro-1,5-indanedione (HIP) and 3aα-H-4α(3′-propionic acid)-5α-hydroxy-7aβ-methylhexahydro-1-indanone (5OH-HIP) by *Rhodococcus equi*. Adapted from [35], [46]–[47]. Numbers represent the following proposed enzymatic steps of β-oxidation of HIP: 1) ATP dependent HIP-CoA transferase, 2) HIP-CoA 5-reductase, 3) acyl-CoA dehydrogenase, 4) 2-enoyl-CoA hydratase, 5) 3-hydroxyacyl-coA dehydrogenase, 6) 3-ketoacyl-CoA thiolase. Dashed lines indicate multiple steps.

In addition to its pathogenic life-style, *R. equi* also thrives as a soil-dwelling microorganism capable of rapid growth in soil and manure using steroids, such as cholesterol, as sole carbon and energy sources [32]–[34]. Microbial steroid degradation of cholesterol proceeds via the formation of 4-androstene-3,17-dione (AD), methylhexahydroindane-1,5-dione propionate (HIP; 3aα-H-4α(3′-propionic acid)-7aβ-methylhexahydro-1,5-indanedione) and 5-hydroxy-methylhexahydro-1-indanone propionate (5OH-HIP) as pathway intermediates (Fig. 1) [35]–[36]. The cholesterol catabolic pathway has been implicated in the pathogenicity of *M. tuberculosis* H37Rv [36]–[39]. Inactivation of the Mce4 cholesterol transporter in *R. equi* RE1, however, did not reveal an essential role of cholesterol catabolism in *R. equi* macrophage survival [34], [40]. Transposon mutagenesis had previously defined a subset of genes required for the survival of *M. tuberculosis* in murine macrophages. Amongst several others, *rv3551* and *rv3552* were predicted to be essential for the survival of *M. tuberculosis* H37Rv *in vitro* in macrophages [41]. Interestingly, *rv3551* and *rv3552* are part of the cholesterol catabolic gene cluster ([36]; Fig. S1). The close phylogenetic relationship between *M. tuberculosis* and *R. equi* prompted us to hypothesize that the predicted critically important genes of the cholesterol catabolic pathway in *M. tuberculosis* H37RV also are important for the pathogenicity of *R. equi* RE1. In this study, we identified the orthologs of *rv3551* and *rv3552*, designated *ipdA* and *ipdB*, respectively, within the cholesterol catabolic gene cluster of *R. equi* 103S. The Δ*ipdAB* mutant of *R. equi* RE1 was impaired in growth on the steroid catabolic pathway intermediates AD and 5OH-HIP. We also observed that RE1Δ*ipdAB* was attenuated *in vitro* in macrophages. RE1Δ*ipdAB* could be safely administered to 2–5 week-old foals intratracheally and oral immunization provided a substantial protection against *R. equi* infection. The data suggests that genes important for methylhexahydroindanone propionate (HIP, 5OH-HIP) degradation, as part of the steroid catabolic pathway, are promising targets for the development of a live-attenuated vaccine against *R. equi* infections.

## Results

### The *R. equi* 103S genome encodes two sets of genes orthologous to *rv3551* and *rv3552* of *M. tuberculosis* H37Rv

Bioinformatic analysis of the sequenced genome of *R. equi* 103S [42] revealed the presence of a cholesterol catabolic pathway (Fig. S1). Within the cholesterol catabolic gene cluster, two genes, i.e. *ipdA* (*REQ_07170*) and *ipdB* (*REQ_07160*), encode proteins that are highly similar to Rv3551 (69% identity) and Rv3552 (67% identity) of *M. tuberculosis* H37Rv, respectively. The similarities of IpdA and IpdB are comparable to those observed between other homologous proteins of the cholesterol catabolic gene clusters of *R. equi* 103S and *M. tuberculosis* H37Rv (Table S1). The operonic structure of *rv3551* and *rv3552* in strain H37Rv was conserved in *R. equi* 103S (Fig. S1). Unlike H37Rv, the genome of *R. equi* 103S encoded a second set of paralogous proteins, designated IpdA2 and IpdB2, respectively, with highest protein sequence similarities to IpdA (55% identity) and IpdB (51% identity), respectively. This second set of genes, designated *ipdA2* (*REQ_00850*) and *ipdB2* (*REQ_00860*), was located outside of the cholesterol catabolic gene cluster and, unlike the *ipdAB* operon, was not clustered with an *echA20* paralog.

IpdA carries the PF01144 signature motif of heterodimeric coenzyme A transferases (http://pfam.sanger.ac.uk; [43]) as well as the COG1788 signature (http://www.ncbi.nlm.nih.gov/Structure/cdd/cdd.shtml) of AtoD, the α subunit of acetoacetyl-CoA transferase of *E. coli*. Moreover, IpdB contained the COG2057 signature motif of AtoA, the β subunit of acetoacetyl-CoA transferase of *E. coli*. Highest overall amino acid sequence similarity of IpdA and IpdB with characterized proteins in databases was observed with ORF1 (41% identity) and ORF2 (36% identity) of *Comamonas testosteroni* TA441, representing the putative α and β subunits of a CoA-transferase, respectively, involved in testosterone catabolism [44]. Mutational analysis in *C. testosteroni* TA441 suggested that *ORF1* is probably involved in the steroid degradation pathway at a step after ring cleavage into HIP and 2-hydroxyhexa-2,4-dienoic acid (Fig. 1; [44]). Since *tesE*, *tesF* and *tesG* are thought to encode the enzymes necessary to degrade 2-hydroxyhexa-2,4-dienoic acid [45], *ORF1* is likely to play a role in HIP degradation. Thus, we hypothesized that IpdA and IpdB of *R. equi* most likely constitute the α-subunit and β-subunit, respectively, of a heterodimeric coenzyme A transferase involved in steroid catabolism, more specifically in methylhexahydroindanone propionate degradation.

### *R. equi* mutant strains RE1Δ*ipdAB* and RE1Δ*ipdAB*Δ*ipdA2B2* are impaired in steroid catabolism

To substantiate the predicted roles of *ipdAB* and *ipdA2B2* in steroid catabolism, we constructed *R. equi* unmarked gene deletion mutant strains RE1Δ*ipdAB*, RE1Δ*ipdA2B2* and RE1Δ*ipdAB*Δ*ipdA2B2* using the two-step homologous recombination strategy with 5-fluorocytosine counter-selection [34]. Deletion of the target genes *ipdAB* and/or *ipdA2B2* was confirmed by PCR for all three mutant strains (Table S2, data not shown). PCR amplicons of the expected sizes were obtained for RE1Δ*ipdAB* mutant (296 bp), RE1Δ*ipdA2B2* (123 bp) and RE1Δ*ipdAB*Δ*ipdA2B2* (296 bp and 123 bp, respectively). Analyses of the upstream and downstream regions of the deleted loci by PCR further confirmed genuine gene deletions and the absence of aberrant genomic rearrangements for all three mutants (Table S2). The presence of *vapA* as a marker for the virulence plasmid was also confirmed by PCR in each of the mutants (Table S2).

**Figure 2:**
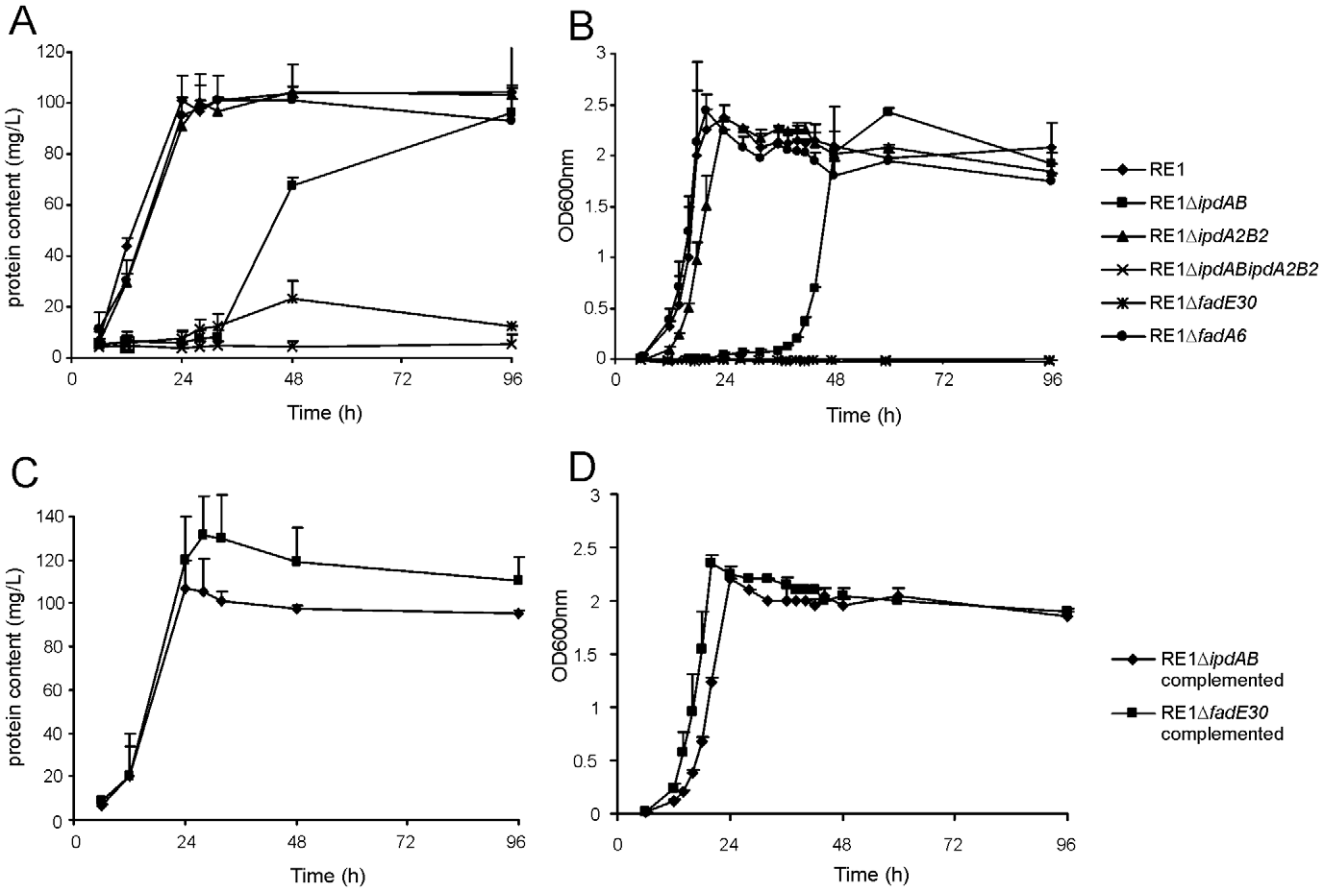
Growth curves of wild type, mutant and complemented mutant strains of *R. equi* RE1 in mineral medium supplemented with 4-androstene-3,17-dione (AD) or 3aα-H-4α(3′-propionic acid)-5α-hydroxy-7aβ-methylhexahydro-1-indanone (5OH-HIP) as a sole carbon and energy source. Panels A and B show growth curves of wild type strain (diamonds) and mutants strains RE1Δ*ipdAB* (squares), RE1Δ*ipdA2B2* (triangles), RE1Δ*ipdAB*Δ*ipdA2B2* (crosses), RE1Δ*fadE30* (asterisks) and RE1Δ*fadA6* (circles) in MM+AD and MM+5OH-HIP, respectively. Panels C and D show growth curves of complemented strains of RE1Δ*ipdAB* (diamonds) and RE1Δ*fadE30* (squares) in MM+AD and MM+5OH-HIP, respectively. Curves represent averages of two independent experiments. Error bars represent standard deviations. Media with AD are turbid; therefore culture protein content was measured instead of optical density.

The growth of all three mutant strains on standard acetate mineral media was comparable to wild type strain RE1 (data not shown). Wild type strain RE1 also showed good growth on the steroid substrate AD as a sole carbon and energy source. By contrast, mutant strain RE1Δ*ipdAB* was severely impaired in growth on AD (Fig. 2A). RE1Δ*ipdAB* displayed an extensive lag-phase in growth of more than 24 h compared to wild type strain RE1. This growth phenotype of RE1Δ*ipdAB* was fully complemented following the introduction of a 4,453 bp DNA fragment carrying wild type *ipdAB* under its native promoter (Table S2), restoring growth on AD to levels comparable to the wild type (Fig. 2C). Since RE1Δ*ipdAB*Δ*ipdA2B2* showed complete blockage of growth on AD, the observed growth of RE1Δ*ipdAB* following the lag-phase appeared to be due to the presence of the paralogous gene set *ipdA2B2* partly complementing the *ipdAB* mutation (Fig. 2A). RE1Δ*ipdA2B2* on the other hand was not impaired in growth on AD and grew comparably to wild type strain, indicating that *ipdAB*, located within the cholesterol gene cluster, is the dominant *ipd* gene set involved in steroid catabolism.

**Figure 3:**
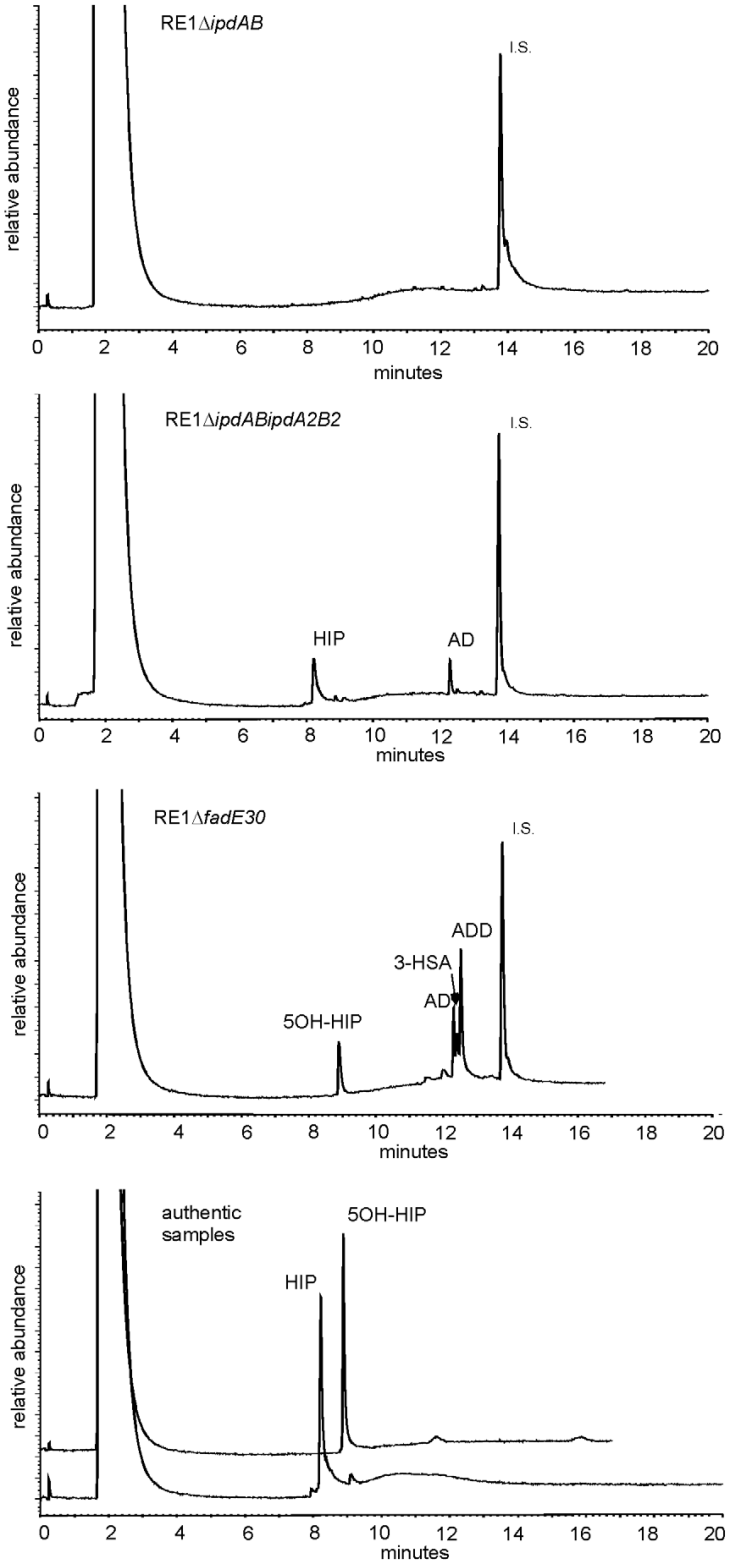
Gas chromatography profiles showing the formation of methylhexahydroindanone propionate intermediates during whole cell biotransformations of 4-androstene-3,17-dione (AD) by mutant strains of *R. equi* RE1 at T = 120 hours. Methylhexahydroindane-1,5-dione propionate (HIP) and 5-hydroxy-methylhexahydro-1-indanone propionate (5OH-HIP) accumulation in cell cultures of RE1Δ*ipdABΔipdA2B2* and RE1Δ*fadE30*, respectively. No accumulation is observed in cell cultures of RE1Δ*ipdAB*. Lower panel shows the GC profiles of HIP (200 mg/L) and 5OH-HIP (50 mg/L) as authentic samples. Abbreviations: 1,4-androstadiene-3,17-dione (ADD), 3-hydroxy-9,10-secoandrost-1,3,5(10)-triene-9,17-dione (3-HSA), progesterone (50 mg/L) internal standard (I.S.).

Next, we investigated whether *ipdAB* and *ipdA2B2* were involved in the catabolism of one of the predicted methylhexahydroindanone propionate intermediates of steroid degradation, i.e. 5OH-HIP (Fig. 2B). The growth phenotype of the mutants in the presence of 5OH-HIP as the sole carbon and energy source was comparable to those observed for AD: an extensive lag-phase in bacterial growth on 5OH-HIP was observed for strain RE1Δ*ipdAB*, whereas hardly any impairment in growth was observed for RE1Δ*ipdA2B2*. Finally, mutant strain RE1Δ*ipdABipdA2B2* displayed no growth on 5OH-HIP at all. To further substantiate the predicted involvement of *ipdAB* and *ipdA2B2* in methylhexahydroindanone propionate degradation, we performed whole cell biotransformation experiments with cell cultures grown in mineral acetate medium and incubated with AD. Wild type strain RE1 fully degraded AD (0.5 g/l) within 24 hours without the accumulation of HIP or 5OH-HIP (data not shown). Accumulation of HIP from AD, however, was observed for mutant strain RE1Δ*ipdAB*Δ*ipdA2B2* (Fig. 3). A temporary accumulation of HIP, 24 hours after the addition of AD, was also detected in biotransformations with strain RE1Δ*ipdAB* (data not shown). HIP formed by RE1Δ*ipdAB* was, however, fully degraded after 120 hours of incubation (Fig. 3), consistent with its growth phenotypes on AD and 5OH-HIP. Thus, the *ipdAB* genes, encoding a heterodimeric CoA transferase in *R. equi* RE1, are important for growth on steroids and fulfil a role in the lower part of the steroid catabolic pathway, more specifically in methylhexahydroindanone propionate degradation.

### Inactivation of genes involved in methylhexahydroindanone propionate catabolism attenuates *R. equi* RE1 infection of macrophages

**Figure 4:**
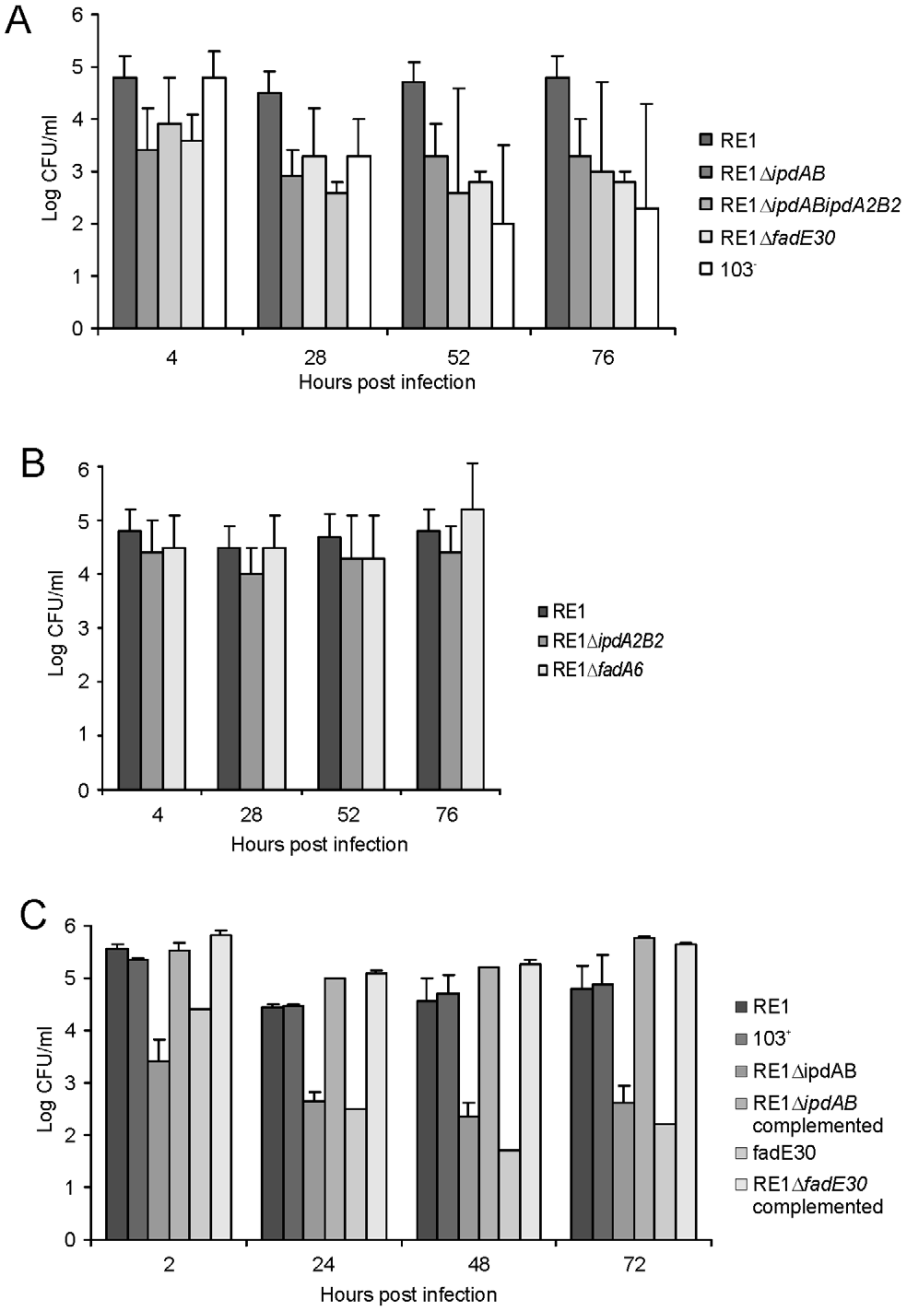
Macrophage infection assays of the human monocyte cell line U937 with *R. equi* strains. Macrophage cell suspensions were infected with wild type virulent strain *R. equi* RE1 or mutant strains RE1Δ*ipdAB*, RE1Δ*ipdA2B2*, RE1Δ*ipdAB*Δ*ipdA2B2*, RE1Δ*fadE30* and RE1Δ*fadA6*. The numbers of intracellular bacteria were determined by plate counts in duplicate following macrophage lysis. The data represents the averages for at least three independent experiments. Error bars represent standard deviations. Panel A shows the results for attenuated mutant strains RE1Δ*ipdAB*, RE1Δ*ipdAB*Δ*ipdA2B2* and RE1Δ*fadE30*. Avirulent strain *R. equi* 103^-^ was used as a control. Panel B shows the results for non-attenuated mutant strains RE1Δ*ipdA2B2* and RE1Δ*fadA6*. Statistically, mutant strains RE1Δ*ipdAB* (P<0.02), RE1Δ*ipdAB*Δ*ipdA2B2* (P<0.01) and RE1Δ*fadE30* (P<0.01) were significantly attenuated compared to parent strain RE1. Panel C shows the results (duplicates) with complemented mutant strains of RE1Δ*fadE30* and RE1Δ*ipdAB*. Wild type RE1, strain 103^+^, and mutant strains RE1Δ*ipdAB* and RE1Δ*fadE30* were included as controls.

To investigate whether the *ipdAB* genes of *R. equi* RE1 are important for survival in macrophages, analogously to the predicted important role of *rv3551* and *rv3552* in *M. tuberculosis* H37Rv [41], *in vitro* macrophage infection assays were performed. Macrophage infection experiments showed that strain RE1Δ*ipdAB* and strain RE1Δ*ipdAB*Δ*ipdA2B2* were significantly attenuated, comparable to the avirulent *R. equi* strain 103^-^ lacking the virulence plasmid [10] (Fig. 4A). Control experiments with virulent wild type strain *R. equi* RE1 showed that the parent strain was able to infect macrophages (Fig. 4A). Inactivation of *ipdAB* was sufficient to significantly impair macrophage infection by *R. equi* RE1 and additional deletion of *ipdA2B2* had no further attenuating effect (Fig. 4A). Consistent with this result, inactivation of *ipdA2B2* alone did not result in attenuation, indicating that *ipdAB* is the dominant gene set involved in *R. equi* RE1 pathogenicity (Fig. 4B). The attenuation of RE1Δ*ipdAB* was fully complemented by the introduction of wild type *ipdAB* (Fig. 4C), excluding the possibility that the attenuation was due to a mutation unrelated to *ipdAB*.

To investigate whether other genes with a role in steroid catabolism are important for macrophage infection by *R. equi* RE1 we constructed additional gene deletion mutants. We chose to inactivate two other genes that were located in close proximity to *ipdAB* within the cholesterol catabolic gene cluster and had been predicted as important for survival of *M. tuberculosis* H37Rv in macrophages, i.e. *fadE30* (*REQ_07030*) and *fadA6* (*REQ_07060*) (Fig. S1; [36], [41]). Mutant strains RE1Δ*fadA6* and RE1Δ*fadE30* were subsequently tested for growth on AD and 5OH-HIP as sole carbon and energy sources. RE1Δ*fadE30* was severely impaired in growth on AD and growth on 5OH-HIP was fully blocked (Fig. 2). The growth phenotype of RE1Δ*fadE30* was fully complemented following the introduction of wild type *fadE30* under its native promoter (Table S2), restoring growth on AD and 5OH-HIP to levels comparable to wild type (Fig. 2C and 2D). Consistent with the growth phenotypes of RE1Δ*fadE30*, cell cultures of mutant strain RE1Δ*fadE30* accumulated 5OH-HIP during biotransformation of AD (Fig. 3). Thus, *fadE30* plays an essential role in steroid catabolism at the level of methylhexahydroindanone propionate degradation. By contrast, RE1Δ*fadA6* was not affected and able to rapidly grow on both 5OH-HIP and AD, comparable to parent strain RE1 (Fig. 2). This suggests that *fadA6* of *R. equi* RE1 is not essential for AD and 5OH-HIP catabolism. However, further analysis revealed that the genome of *R. equi* 103S codes for an apparent paralog of FadA6 (*REQ_21310*) with 70% protein sequence identity. The possibility that *fadA6* of RE1 is involved in steroid catabolism, but is not essential due to the presence of the gene paralog, therefore cannot be excluded at this point.

Macrophage infection assays revealed that strain RE1Δ*fadE30* was significantly attenuated, comparable to that of the attenuated mutant strains RE1Δ*ipdAB* and RE1Δ*ipdAB*Δ*ipdA2B2*, and the avirulent control strain *R. equi* 103^-^ (Fig. 4A). The attenuation of RE1Δ*fadE30* could be fully reversed by the introduction of wild type *fadE30*, indicating that attenuation was solely due to *fadE30* gene inactivation (Fig. 4C). Interestingly, RE1Δ*fadA6* was not attenuated and showed survival curves similar to parent strain RE1 (Fig. 4B), consistent with our hypothesis that *R. equi* RE1 mutant strains impaired in growth on methylhexahydroindanone propionate are attenuated.

### Intratracheal challenge of foals revealed *in vivo* attenuation of mutant strain RE1Δ*ipdAB*

**Figure 5:**
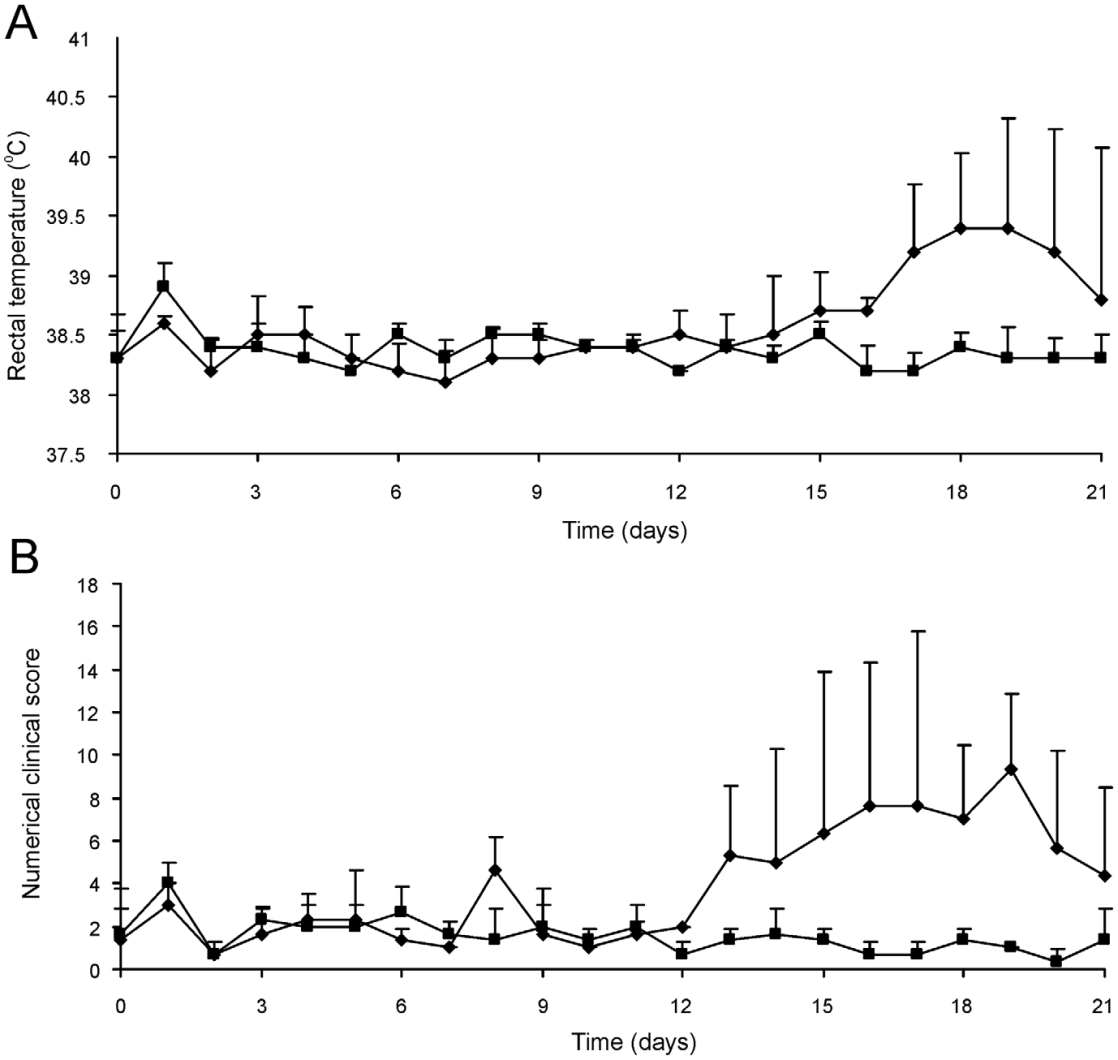
Intratracheal challenge of 3 to 5-week-old foals. Foals (mean of n = 3) were challenged intratracheally with mutant *R. equi* RE1Δ*ipdAB* (7.1×10^6^ CFU; squares) or wild type RE1 (4.3×10^6^ CFU; diamonds). Panel A shows rectal temperatures. Panel B shows numerical clinical scores. Error bars represent standard deviation.

**Figure 6:**
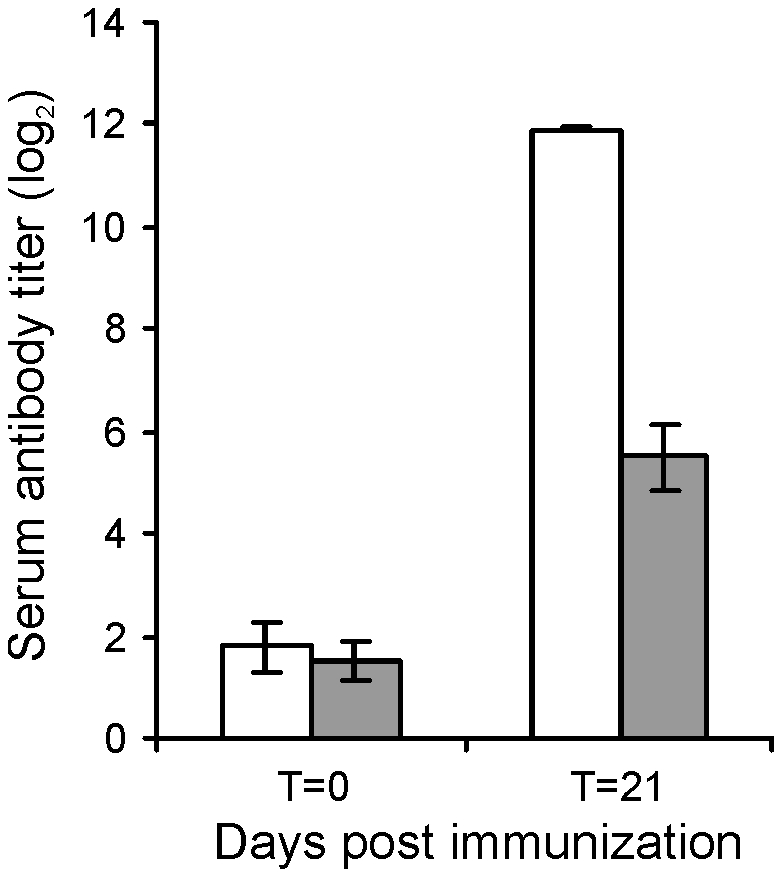
Serum antibody titer against *R. equi* of 3 to 5-week-old foals (n = 3) at day of intratracheal challenge (T = 0 days) and 3-weeks post-challenge (T = 21 days) with mutant *R. equi* RE1Δ*ipdAB* (7.1×10^6^ CFU; grey bars) or wild type RE1 (4.3×10^6^ CFU; white bars). Values represent mean ± standard deviation (error bars).

The attenuated phenotype of RE1Δ*ipdAB* in our *in vitro* macrophage infection model suggested that strain RE1Δ*ipdAB* also might be attenuated in foals. This prompted us to perform an *in vivo* intratracheal challenge experiment in young foals. *In vivo* attenuation of the RE1Δ*ipdAB* mutant was tested in foals aged 3–5 weeks. The foals were equally divided into two groups of three (n = 3). One group was challenged intratracheally with mutant strain RE1Δ*ipdAB* (7.1×10^6^ CFU) and the other group with wild type strain RE1 (4.3×10^6^ CFU) as a control. During a period of 3 weeks post-challenge the foals were clinically scored. None of the foals challenged with RE1Δ*ipdAB* developed signs of respiratory disease and no increase in rectal temperatures of these foals was observed (Fig. 5). By contrast, two out of three foals in the wild type infected group developed severe clinical signs of respiratory disease, coinciding with increased rectal temperatures from 14 days post-challenge onwards. One wild type infected foal showed only mild clinical signs post-challenge. Mean daily weight gains post-challenge were substantially higher for foals challenged with RE1Δ*ipdAB* (27.9 ± 5.2%) compared to those challenged with RE1 (18.9 ± 1.3%). Serum blood analyses revealed that the RE1Δ*ipdAB* mutant strain was able to elicit a substantial serum antibody titer against *R. equi*, although the titers were lower than those observed in foals challenged with strain RE1 (Fig. 6). At 3 weeks post-challenge all foals were euthanized and subjected to a complete post-mortem examination. Foals challenged with wild type strain RE1 had developed typical pyogranulomatous pneumonia from which wild type *R. equi* successfully was re-isolated (Table 1). The lungs of the foals challenged with the mutant strain, on the other hand, did not reveal pneumonic areas and *R. equi* could not be isolated (Table 1). Consistent with these observations, the mean percentage lung-to-body weight of foals challenged with wild type RE1 (2.0 ± 0.6%) was twice as high as those challenged with mutant strain RE1Δ*ipdAB* (1.0 ± 0.06%).

**Table 1:** Pulmonary consolidation and re-isolation of *R. equi* of 3 to 5-week-old foals (n = 3) challenged intratracheally with wild type strain RE1 (4.3×10^6^ CFU) or mutant strain RE1Δ*ipdAB* (7.1×10^6^ CFU).

ChallengeStrain	Foal	Lung weight per total weight (%)	Pulmonary consolidation per lobe (%) a					Isolation of *R. equi* from lung (log_10_ CFU/ml homogenate) b
			Apical left	Apical right	Caudal left	Caudal right	Accessory	
RE1	1	1.4	5	30	5	30	30	4.2 ± 0.57
	2	2.6	10	0	60	40	70	6.8 ± 0.67
	3	1.9	50	70	50	70	90	3.1 ± 2.2
	Mean	2.0	22	33	38	47	63	4.7 ± 1.8
RE1 Δ*ipdAB*	4	1.0	0	0	0	0	0	0
	5	1.1	0	0	0	0	0	0
	6	1.0	0	0	0	0	0	0
	Mean	1.0	0	0	0	0	0	0

a percentage of pulmonary consolidation was determined by an experienced pathologist.

b average value calculated from numbers found in apical, lower caudal, upper caudal and accessory lobes.

### Oral immunization of foals with RE1Δ*ipdAB* provides substantial protection against an intratracheal challenge

**Figure 7:**
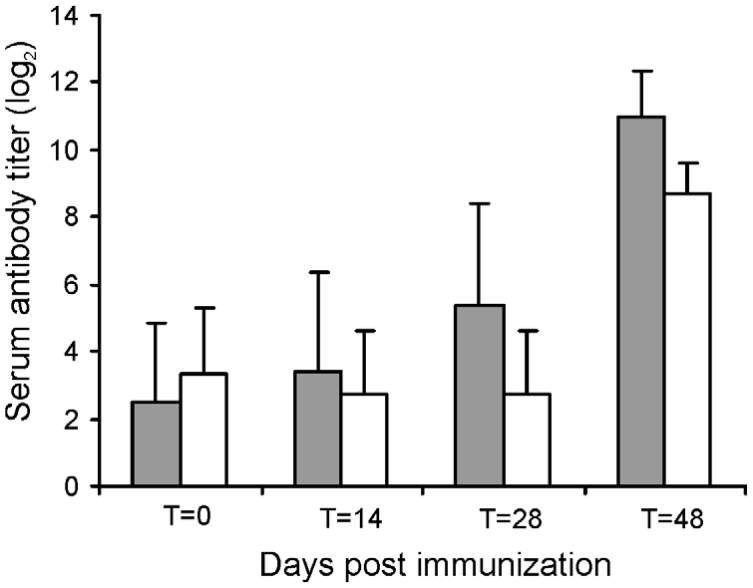
Serum antibody titer against *R. equi* of foals (n = 4) immunized orally (grey bars) at T = 0 and T = 14 days with attenuated *R. equi* strain RE1Δ*ipdAB* (5×10^7^ CFU) and challenged at T = 28 days with *R. equi* strain 85F (5×10^6^ CFU). The serum antibody titer of unvaccinated control foals (n = 4) are shown in white bars. Bars represent mean titers at day of vaccination (T = 0), at day of booster vaccination (T = 14), at day of intratracheal challenge (T = 28) and 20 days post-challenge (T = 48). Error bars represent standard deviation.

The challenge experiments indicated that RE1Δ*ipdAB* was attenuated in young foals and able to induce an immunological response. To test RE1Δ*ipdAB* as a live-attenuated vaccine-candidate in providing protective immunity against an intratracheal challenge with virulent *R. equi*, we performed an immunization experiment. Eight 2 to 4-week-old foals were used for this experiment and divided into two groups of four foals (n = 4). At T = 0 and at T = 14 days (booster) one group was vaccinated orally (1 ml) with strain RE1Δ*ipdAB* (5×10^7^ CFU/animal) and the other group was left as unvaccinated control. After vaccination, all foals remained healthy and no vaccine-related abnormalities were observed. Rectal temperatures remained normal in all foals (data not shown). Strain RE1Δ*ipdAB* could not be re-isolated from rectal swabs, indicating that the mutant strain did not massively colonize the alimentary tract. Serum blood analyses revealed substantial serum antibody titers against *R. equi* following vaccination (Fig. 7). These post-vaccination results were consistent with the results obtained from the challenge experiment and confirmed that mutant strain RE1Δ*ipdAB* was attenuated *in vivo* and can be safely administered to young foals.

**Figure 8:**
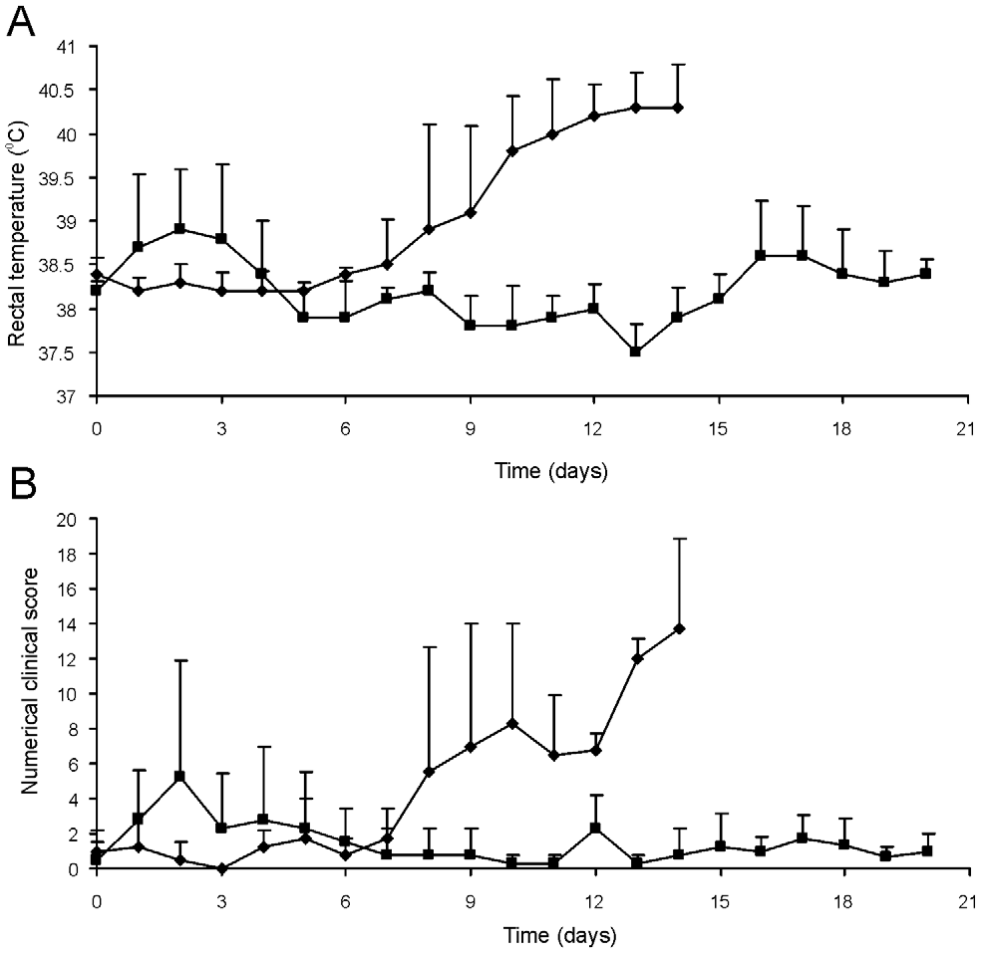
Oral immunization and subsequent intratracheal challenge of foals. Foals (2 to 4-week-old) vaccinated with RE1Δ*ipdAB* (squares) and non-vaccinated controls (diamonds) (mean of n = 4) were challenged intratracheally with virulent strain *R. equi* 85F (5×10^6^ CFU). Panel A shows rectal temperatures. Panel B shows numerical clinical scores. Statistically, rectal temperatures (P<0.005) and clinical scores (P<0.0001) were significantly different in vaccinates compared to the non-vaccinated control foals. Error bars represent standard deviation.

**Figure 9:**
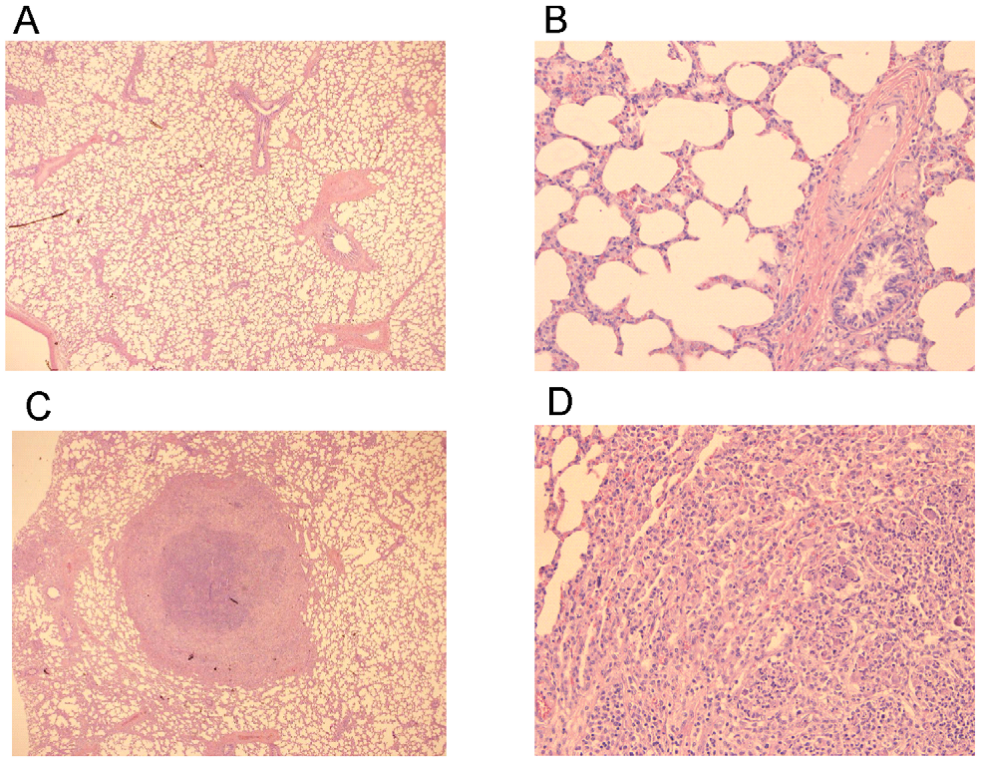
Histopathology of lung tissue of vaccinates versus non-vaccinated foals following intratracheal challenge with wild type *R. equi*. Lung specimen of a vaccinated foal showing normal airways (bronchi and bronchioli), blood vessels and alveoli at (A) 25x and (B) 200x magnification. Typical pyogranuloma (5 mm diameter) observed in lung specimens of non-vaccinated control foals at (C) 25x and (D) 200x magnification. The centre of the pyogranuloma consists of necrotic debris, neutrophils and toxic neutrophils with complete loss of lung architecture.

All foals were subsequently challenged intratracheally with virulent strain *R. equi* 85F (5×10^6^ CFU), displaying strong cytotoxicity [16], two weeks after the booster vaccination (T = 28 days). During a period of 3 weeks post-challenge the foals were clinically scored. Then foals were euthanized and subjected to a complete post-mortem examination with special attention to the lungs and respiratory lymph nodes as well as the gut and associated lymph nodes. All four foals in the control group showed increasing signs of respiratory disease from day 7 to 10 post-challenge onwards (Fig. 8; T = 35–38 days). The control foals were euthanized 14 days post challenge (T = 42 days) for humane reasons. Post-mortem macroscopic and microscopic analysis confirmed pyogranulomatous pneumonia in the control foals with severe pulmonary consolidations from which wild type *R. equi* was re-isolated as identified by PCR (Tables 2 and 3; Fig. 9). Wild type *R. equi* was also isolated in high numbers from swollen mediastinal lymph nodes and in one foal from a caecal lymph node. By contrast, the vaccinated foals had much milder clinical signs or virtually no clinical signs at all (Fig. 8). Two vaccinated foals remained completely healthy and post-mortem macroscopic analysis did not reveal any signs of pyogranulomatous pneumonia. Two other vaccinates had locally developed pyogranulomatous pneumonia with pulmonary consolidations in the accessory and caudal lobes from which wild type *R. equi* was isolated (Tables 2 and 3). Overall, the numbers of wild type *R. equi* isolated from the lungs of the vaccinated foals were substantially lower than those found in the control group. We conclude that vaccination of young foals with strain RE1Δ*ipdAB* is safe and induces a substantial protective immunity against a severe intratracheal challenge with a virulent *R. equi* strain.

**Table 2:** Lung weights and percentage pulmonary consolidation per lobe of vaccinated and unvaccinated (control) 2 to 4-week-old foals (n = 4).

Foal	Age at challenge (weeks)	Lung weight per total weight (%)	Pulmonary consolidation per lobe (%) a				
			Apical left	Apical right	Caudal left	Caudal right	Accessory
Vaccinate 1	8	1.4	0	0	50	5	40
Vaccinate 2	8	1.0	1^b^	1^b^	1^b^	1^b^	0
Vaccinate 3	7	1.2	3^b^	0	10	10	70
Vaccinate 4	7	1.1	0	0	0	0	0
Mean vaccinates		1.2	1	0	15	4	28
Control 1	8	2.6	10	60	80	80	90
Control 2	8	2.0	0	50	40	70	70
Control 3	7	3.4	10	60	70	70	90
Control 4	6	2.9	0	10	40	80	100
Mean controls		2.7	5	45	58	75	88

Foal	*R. equi* 85F (log_10_ CFU/ml homogenate)				
	Apical lung lobe	Upper caudal lung lobe	Lower caudal lung lobe	Accessory Lobe	Mean Lung
Vaccinate 1	0	0	4.26	5.18	2.36
Vaccinate 2	0	0	0	0	0
Vaccinate 3	0	5.13	0	6.15	2.82
Vaccinate 4	0	0	0	0	0
Mean vaccinates	0.00	1.28	1.07	2.83	1.30
Control 1	8.20	9.19	8.97	8.90	8.82
Control 2	6.75	7.02	7.11	6.96	6.96
Control 3	8.81	8.89	9.08	8.86	8.91
Control 4	5.19	6.13	5.95	6.38	5.91
Mean controls	7.24	7.81	7.78	7.78	7.65

Foals were vaccinated orally at T = 0 and T = 2 weeks with RE1Δ*ipdAB* (5×10^7^ CFU/animal). Foals were challenged intratracheally at T = 4 weeks with the highly virulent strain *R. equi* 85F (5×10^6^ CFU/animal). Statistically, pulmonary consolidation was significantly different in vaccinates compared to the non-vaccinated control foals (P<0.01).

a percentage of pulmonary consolidation was determined by an experienced pathologist.

b consolidated, but not pyogranulomatous.

**Table 3:**
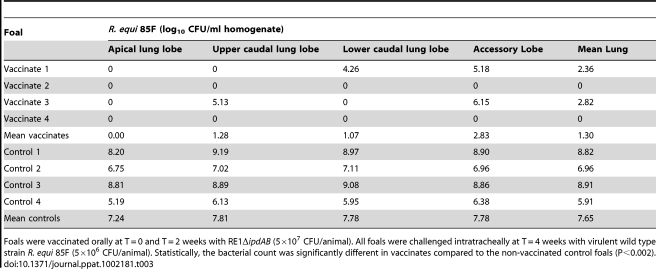
Quantitative re-isolation of *R. equi* 85F from lung lobes of vaccinated and unvaccinated (control) 2 to 4-week-old foals (n = 4).

Foals were vaccinated orally at T = 0 and T = 2 weeks with RE1Δ*ipdAB* (5×10^7^ CFU/animal). All foals were challenged intratracheally at T = 4 weeks with virulent wild type strain *R. equi* 85F (5×10^6^ CFU/animal). Statistically, the bacterial count was significantly different in vaccinates compared to the non-vaccinated control foals (P<0.002).

## Discussion

The current study identified the cholesterol catabolic gene cluster in *R. equi* and showed that *ipdAB* and *fadE30* located within this cluster are important for the pathogenicity of *R. equi* RE1. Interestingly, *R. equi* RE1 mutants that displayed attenuated phenotypes in an *in vitro* macrophage infection assay were also impaired in steroid catabolism, i.e. RE1Δ*ipdAB*, RE1Δ*ipdAB*Δ*ipdA2B2* and RE1Δ*fadE30*. Conversely, mutants that had AD growth phenotypes comparable to wild type strain RE1, i.e. RE1Δ*ipdA2B2* and RE1Δ*fadA6*, were not attenuated. Both *fadE30* and *ipdAB* were also shown to be important for 5OH-HIP catabolism. Biochemical and physiological studies previously showed that the degradation of the propionate moiety of HIP and 5OH-HIP likely occurs via a cycle of β-oxidation [35], [46]–[47] (Fig. 1). ATP-dependent CoA activation was suggested to be the first step in the degradation of HIP in *R. equi* ATCC14887 [46]. Protein sequence analysis revealed that IpdA and IpdB represent the α and β-subunit of a heterodimeric CoA-transferase. The heterodimeric CoA-transferase encoded by *ipdAB* thus might be involved in the removal of the propionate moiety of methylhexahydroindanone propionate intermediates (i.e. HIP, 5OH-HIP) by β-oxidation during steroid degradation (Fig. 1, step 1). Consistent with such a role, HIP accumulation was observed in biotransformation experiments with cell cultures of RE1Δ*ipdABΔipdA2B2* incubated with AD (Fig. 3). FadE30 belongs to the family of acyl-CoA dehydrogenases and might catalyze the second step in the β-oxidation cycle that removes the propionate moiety following CoA activation by IpdAB, i.e. the dehydrogenation of 5OH-HIP-CoA (Fig. 1, step 3). Accumulation of 5OH-HIP indeed was observed in biotransformation experiments with cell cultures of RE1Δ*fadE30* incubated with AD (Fig. 3). Interestingly, *ipdA* and *ipdB* appear to be part of an operon encompassing *echA20* (Fig. S1), encoding a putative enoyl-coA hydratase that might catalyse the subsequent step in the β-oxidation cycle during the degradation of the propionate moiety (Fig. 1, step 4). However, functions of *ipdAB*, *fadE30* and *echA20* further down in the degradation pathway of these compounds cannot be excluded and need further investigation.

A second set of paralogous genes, designated *ipdA2* and *ipdB2*, was additionally identified in *R. equi* RE1 which do not play an important role in AD or 5OH-HIP catabolism. Still, *ipdA2B2* are involved in growth on AD and 5OH-HIP, since *ipdA2B2* are able to support the growth of mutant strain RE1Δ*ipdAB* on AD and 5OH-HIP, albeit after an extensive lag-phase (Fig. 2). The data suggests that the primary role of *ipdA2B2* is not in AD or 5OH-HIP catabolism, but that they are recruited in the Δ*ipdAB* mutant, perhaps through a genetic mutation. Protein sequence similarities between IpdA and IpdA2 and between IpdB and IpdB2 are relatively low, which suggests that IpdAB and IpdA2B2 are related proteins, but have different physiological functions. This is further supported by the genomic location of *ipdA2* and *ipdB2* in a region distant from the cholesterol catabolic gene cluster and with no apparent clustering of steroid genes. Consistently, *ipdA2B2* does not appear to be involved in pathogenesis. Due to the likely different physiological function of *ipdA2B2* in *R. equi* these genes may be expressed differently relative to *ipdAB*, or even not at all, during *R. equi* infection.

Overall, our results strongly imply that the pathogenicity of *R. equi* correlates with the steroid catabolic pathway, in particular with methylhexahydroindanone propionate (HIP, 5OH-HIP) degradation. Several other examples of virulence-associated genes important for microbial steroid ring degradation have been reported. The *kshA* and *kshB* genes of *M. tuberculosis* H37Rv, for example, were shown to be essential for pathogenicity of H37Rv [39]. These genes encode the two-component iron-sulfur protein 3-ketosteroid 9α-hydroxylase, which is a key-enzymatic step in microbial steroid ring opening [48]. The steroid ring-cleaving dioxygenase HsaC, catalyzing the further breakdown of steroids towards methylhexahydroindanone propionate pathway intermediates, also contributes to the pathogenicity of *M. tuberculosis* H37Rv [38]. We do not yet understand why genes of the steroid catabolic pathway are important for the pathogenicity of *R. equi*. Considering that many steroids are known to have immuno-regulatory properties, steroids could play an important role during *R. equi* infection. *In vivo*, β-androstenes, such as 3β-hydroxy-5-androstene-17-one (DHEA) and 5-androstene-3β,17β-diol, have been associated with immune-homeostasis during bacterial infection [49]. Thus, *ipdAB*, *fadE30* and other genes involved in steroid ring degradation may help *R. equi* to disrupt the immune-homeostasis in a yet unknown way, favouring infection of the macrophage. Intriguingly, attenuated mutant strains RE1Δ*ipdAB*, RE1Δ*ipdAB*Δ*ipdA2B2* and RE1Δ*fadE30* consistently showed significantly lower bacterial counts in our macrophage infection assay at T = 4 h post-infection (Fig. 4A) compared to wild type strains RE1 and avirulent strain 103^−^, which suggests that the attenuated mutants are affected in processes that occur early in the infection. Whether these processes are involved in immune-homeostasis or are related to some other process, such as impaired adherence or uptake of *R. equi* by the macrophage, remains to be elucidated.

It is noteworthy to mention that, for reasons unknown, wild type *R. equi* strains RE1 and 103^+^ do not appear to replicate well in the human macrophage cell line U937 when compared to the replication of wild type *R. equi* in murine or equine primary macrophages.

A subset of genes of the cholesterol gene cluster present in *Mycobacterium smegmatis* mc^2^155, designated the *kstR2* regulon, was recently shown to be controlled by the TetR-type transcriptional regulator *kstR2* [50]. An apparent orthologue of *kstR2* of *M. smegmatis* mc^2^155 was also found present in the cholesterol gene cluster of *R. equi* 103S, encoding a protein with 56% amino acid sequence identity and located between *fadE30* and *fadA6* (Fig. S1). Interestingly, the *fadA6*, *fadE30* and *ipdAB* orthologues in *M. smegmatis* mc^2^155 all are part of the *kstR2* regulon [50]. Most likely, the *kstR2* regulon of *M. smegmatis* mc^2^155 is involved in methylhexahydroindanone propionate catabolism. The presence of a putative *kstR2* regulon in *R. equi* 103S raises the intriguing question whether all genes belonging to this regulon are important for *R. equi* pathogenicity.

Several vaccination strategies have been explored to date in an attempt to prevent infection by the opportunistic horse pathogen *R. equi*. So far, these have not resulted in the development of a safe and effective vaccine against *R. equi* infection. Indeed, protection has been observed when wild type virulent *R. equi* was administrated orally [51]–[53]. However, this vaccination approach cannot be used due to the high risk of provoking disease and contamination of the environment. Immunization procedures using avirulent (plasmid-less) or killed *R. equi* cells, on the other hand, do not induce a protective immune response [52] and underline the importance of developing a live-attenuated vaccine strain. The administration of specific hyperimmune plasma currently has been the only method providing a positive effect in avoiding foals of an endemic farm to develop *R. equi* pneumonia [54]–[56]. The method, however, is expensive, labour intensive and not consistently effective [57]–[59]. Our strategy targeted genes in the cholesterol catabolic gene cluster of *R. equi* to develop a live-attenuated vaccine. Our data revealed that RE1Δ*ipdAB* is a highly promising candidate for a live-attenuated vaccine strain providing substantial protective immunity. Full immunity following oral immunization with RE1Δ*ipdAB* was not yet observed in the vaccination experiment, since two foals showed mild signs of pneumonic disease following a severe challenge with *R. equi* 85F (Tables 2 and 3). However, re-isolation of wild type *R. equi* was several log_10_ fold lower in lungs of immunized foals compared to those of non-vaccinated controls (Table 3), strongly suggesting that protection had not yet fully developed. Further optimization of the vaccination protocol to increase its efficacy, as well as field trials, is currently on the way to develop the first safe and effective live-attenuated vaccine against *R. equi* infection in young foals.

The incidence of *R. equi* infection in humans has increased markedly with human immunodeficiency virus (HIV) infection, as well as with the development of organ transplantations and chemotherapy for malignancies [1], [60]–[61]. The infection mortality rate is still high (20–25%), especially for AIDS patients (50–55%), and disease relapses are common [60], [62]. The steroid catabolic pathway of *R. equi* therefore may provide interesting novel targets for drug development to treat *R. equi* infection in humans, as many of the catabolic enzymes have no human homolog.

## Materials and Methods

### Culture media and growth conditions

R. equi RE1 was isolated from a foal with pyogranulomatous pneumonia in the Netherlands in September 2007 [34]. Strains R. equi 103^+^ [14], R. equi 103^−^ [63] and R. equi 85F [52], [64] have been previously described. R. equi cell cultures were routinely grown at 30°C (200 rpm) in Luria-Bertani (LB) medium consisting of Bacto-Tryptone, Yeast Extract and 1% NaCl, or mineral acetate medium (MM-Ac) containing K2HPO4 (4.65 g/l), NaH2PO4·H2O (1.5 g/l), sodium acetate (2 g/l), NH4Cl (3 g/l), MgSO4·7H2O (1 g/l), thiamine (40 mg/l, filter sterile), and Vishniac stock solution (1 ml/l). Vishniac stock solution was prepared as follows (modified from Vishniac and Santer [65]): EDTA (10 g/l) and ZnSO4.7H2O (4.4 g/l) were dissolved in distilled water (pH 8.0 using 2 M KOH). Then, CaCl2.2 H2O (1.47 g/l), MnCl2.7 H2O (1 g/l), FeSO4.7 H2O (1 g/l), (NH4)6 Mo7O24.4 H2O (0.22 g/l), CuSO4.5 H2O (0.315 g/l) and CoCl2.6 H2O (0.32 g/l) were added in that order maintaining pH at 6.0 and finally stored at pH 4.0. For growth on 4-androstene-3,17-dione (AD, 0.5 g/l) or 3aα-H-4α(3′-propionic acid)-5α-hydroxy-7aβ-methylhexahydro-1-indanone (5OH-HIP, 1 g/l) as sole carbon and energy source sodium acetate was omitted from the medium. Stock solutions of 5OH-HIP (100 mg/ml), prepared by dissolving 100 mg 3aα-H-4α(3′-propionic acid)-5α-hydroxy-7aβ-methylhexahydro-1-indanone-δ-lactone (HIL) in 1 ml 0.5M NaOH, and AD (50 mg/ml in dimethylsulfoxide (DMSO)) were used. Cultures (50 ml) were inoculated (1∶100) using pre-cultures grown in MM-Ac. Growth was followed by regular turbidity measurements (OD600nm). Turbidity measurements of AD grown cultures could not be accurately determined due to high background of the AD suspension. Protein content of the culture was therefore used as a measure for biomass formation and was determined as follows. A sample (0.5 ml) of the culture was pelleted by centrifugation (5 min 12,000 x g), thoroughly resuspended in 0.1 ml 1 M NaOH and boiled for 10 min. Then, 0.9 ml distilled water was added and the suspension was vortexed. An aliquot of 100 µl was mixed with 300 µl of distilled water and 100 µl of protein assay reagent (BioRad). Protein content of the sample was determined using bovine serum albumin (BSA) as a standard as described by the manufacturer. For growth on solid media Bacto-agar (15 g/l; BD) was added. 5-Fluorocytosine stock solution (10 mg/ml) was prepared in distilled water, dissolved by heating to 50°C, filter-sterilized and added to autoclaved media.

### Cloning, PCR and genomic DNA isolation

*Escherichia coli* DH5α was used as host for all cloning procedures. Restriction enzymes were obtained from Fermentas GmbH. Chromosomal DNA of cell cultures was isolated using the GenElute Bacterial Genomic DNA Kit (Sigma-Aldrich) according to the instructions of the manufacturer. PCR was performed in a reaction mixture (25 µl) consisting of Tris-HCl (10 mM, pH 8), 1x standard polymerase buffer, dNTPs (0.2 mM), DMSO (2%), PCR primers (10 ng/µl each) and High-Fidelity DNA polymerase enzyme (Fermentas) or Pwo DNA polymerase (Roche Applied Science). For colony PCR, cell material was mixed with 100 µl of chloroform and 100 µl of 10 mM Tris-HCl pH 8, vortexed vigorously and centrifuged (2 min, 14,000 x g). A sample of the upper aqueous phase (1 µl) was subsequently used as template for PCR. A standard PCR included a 5 min 95°C DNA melting step, followed by 30 cycles of 45 sec denaturing at 95°C, 45 sec annealing at 60°C and 1–3 min elongation at 72°C. The elongation time used depended on the length of the expected PCR amplicon, taking 1.5 min/1 kb as a general rule.

### Electrotransformation of *R. equi* strains

Cells of *R. equi* strains were transformed by electroporation essentially as described [34]. Briefly, cell cultures were grown in 50 ml LB at 30°C until OD_600_ reached 0.8–1.0. The cells were pelleted (20 min at 4,500 x g) and washed twice with 10% ice-cold glycerol. Pelleted cells were re-suspended in 0.5–1 ml ice-cold 10% glycerol and divided into 200 µl aliquots.

MilliQ-eluted plasmid DNA (5–10 µl; GenElute Plasmid Miniprep Kit, Sigma-Aldrich) was added to 200 µl cells in 2 mm gapped cuvettes. Electroporation was performed with a single pulse of 12.5 kV/cm, 1000Ω and 25 µF. Electroporated cells were gently mixed with 1 ml LB medium (*R. equi*) and allowed to recover for 2 h at 37°C and 200 rpm. Aliquots (200 µl) of the recovered cells were plated onto selective agar medium. *R. equi* transformants were selected on LB agar containing apramycin (50 µg/ml) and appeared after 2–3 days of incubation at 30°C.

### Construction of unmarked gene deletion mutants of *Rhodococcus equi* RE1

Unmarked gene deletion mutants of *R. equi* RE1 were constructed essentially as described previously [34]. All oligonucleotide primers used in the construction of plasmids necessary for the construction of the mutants are shown in Table S2. Plasmid pSelAct-ipd1, for the generation of an unmarked gene deletion of the *ipdAB* operon in *R. equi* RE1, was constructed as follows. The upstream (1,368 bp; primers ipdABequiUP-F and ipdABequiUP-R) and downstream (1,396 bp; primers ipdABequiDOWN-F and ipdABequiDOWN-R) flanking regions of the *ipdAB* genes were amplified by PCR. The obtained amplicons were ligated into *Eco*RV digested pBluescript(II)KS, rendering plasmids pEqui14 and pEqui16 for the upstream and downstream region, respectively. A 1.4 kb *Spe*I/*Eco*RV fragment of pEqui14 was ligated into *Spe*I/*Eco*RV digested pEqui16, generating pEqui18. A 2.9 kb *Eco*RI/*Hind*III fragment of pEqui18, harboring the *ipdAB* gene deletion and its flanking regions, was treated with Klenow fragment and ligated into *Sma*I digested pSelAct suicide vector [34]. The resulting plasmid was designated pSelAct-ipd1 for the construction of *ipdAB* gene deletion mutant *R. equi* Δ*ipdAB*. Complementation of mutant strain RE1ΔipdAB was performed by introduction of a 4.4 kb DNA fragment obtained by PCR using primers ipdABequiContrUP-F and ipdABequiContrDOWN-R. The PCR product obtained was cloned into pSET152 and the resulting construct was introduced into RE1Δ*ipdAB* by electroporation [34].

Mutant strain RE1Δ*ipdA2B2* was constructed by unmarked gene deletion of the *ipdA2B2* operon from *R. equi* RE1 using plasmid pSelAct-ΔipdAB2. Double gene deletion mutant *R. equi* RE1Δ*ipdAB*Δ*ipdA2B2* was made in *R. equi* RE1Δ*ipdAB* mutant strain. Plasmid pSelAct-ΔipdAB2 was constructed as follows. The upstream (1,444 bp; primers ipdAB2equiUP-F and ipdAB2equiUP-R) and downstream (1,387 bp; ipdAB2equiDOWN-F, ipdAB2equiDOWN-R) regions of *ipdAB2* were amplified by PCR using genomic DNA as template (Table S2). The amplicons were ligated into *Sma*I digested pSelAct, resulting in plasmids pSelAct-ipdAB2equiUP and pSelAct-ipdAB2equiDOWN, respectively. Following digestion with *Bgl*II/*Spe*I of both plasmids, a 1,381 bp fragment of pSelAct-ipdAB2equiDOWN was ligated into pSelAct-ipdAB2equiUP, resulting in pSelAct-ΔipdAB2 used for the construction of a Δ*ipdA2B2* gene deletion.

Plasmid pSelAct-fadE30 for the generation of an unmarked gene deletion of *fadE30* in *R. equi* RE1 was constructed as follows. The upstream (1,511 bp; primers fadE30equiUP-F and fadE30equiUP-R) and downstream (1,449 bp; primers fadE30equiDOWN-F and fadE30equiDOWN-R) flanking genomic regions of *fadE30* were amplified by a standard PCR using High Fidelity DNA polymerase (Fermentas GmbH). The obtained amplicons were ligated into the pGEM-T cloning vector (Promega Benelux), rendering pGEMT-fadE30UP and pGEMT-fadE30DOWN. A 1.4 kb *Bcu*I/*Bgl*II DNA fragment was cut out of pGEMT-fadE30DOWN and ligated into *Bcu*I/*Bgl*II linearized pGEMT-fadE30UP, resulting in pGEMT-fadE30. To construct pSelAct-fadE30, pGEMT-fadE30 was digested with *Nco*I and *Bcu*I and treated with Klenow fragment. A 2.9 kb blunt-end DNA fragment, carrying the *fadE30* gene deletion, was ligated into *Sma*I digested pSelAct [34]. The resulting plasmid was designated pSelAct-fadE30 and used for the construction of mutant strain *R. equi* RE1Δ*fadE30*. Complementation of mutant strain RE1Δ*fadE30* was performed by the introduction of a 2.8 kb DNA fragment obtained by PCR using primers fadE30equiUP-F and fadE30Contr-R (Table S2). The PCR product obtained was cloned into *Eco*RV digested pSET152 and the resulting construct was introduced into RE1Δ*fadE30* by electroporation [34].

Plasmid pSelAct-fadA6 for the generation of an unmarked gene deletion of *fadA6* in *R. equi* RE1 was constructed as follows. The upstream (1,429 bp; primers fadA6equiUP-F and fadA6equiUP-R) and downstream (1,311 bp; primers fadA6equiDOWN-F and fadA6equiDOWN-R) flanking genomic regions of *fadA6* were amplified by a standard PCR using High Fidelity DNA polymerase (Fermentas GmbH). The obtained amplicons were ligated into the pGEM-T cloning vector (Promega Benelux), rendering pGEMT-fadA6UP and pGEMT-fadA6DOWN. A 1.4 kb *Spe*I/*Bgl*II DNA fragment was cut out of pGEMT-fadA6UP and ligated into *Spe*I/*Bgl*II linearized pGEMT-fadA6DOWN, resulting in pGEMT-fadA6. To construct pSelAct-fadA6, pGEMT-fadA6 was digested with *Eco*RI and a 2.7 kb DNA fragment, carrying the *fadA6* gene deletion, was ligated into *Eco*RI digested pSelAct [34]. The resulting plasmid was designated pSelAct-fadA6 and used for the construction of mutant strain *R. equi* RE1Δ*fadA6*.

### GC analysis of HIP and 5OH-HIP formation in whole-cell biotransformations of AD

Strains were pre-grown (30°C, 200 rpm) in LB medium (10 ml) overnight and subsequently inoculated (1∶100) in 50 ml MM-Ac and incubated (30°C, 200 rpm) for 36 hours. AD (0.5 ml of 50 mg/ml stock in DMSO) was then added. Samples (0.25 ml) for GC analysis were collected and acidified with 5 µl 10% H_2_SO_4_ at several intervals. Progesterone (10 µl of a 5 mg/ml stock in ethylacetate) was added as an internal standard and samples were subsequently extracted using ethylacetate (1 ml). GC analysis was performed on a GC8000 TOP (Thermoquest Italia, Milan, Italy) equipped with an EC-5 column measuring 30 m by 0.25 mm (inner diameter) and a 0.25 µm film (Alltech, Ill., USA.) and FID detection at 300°C. Chromatographs obtained were analysed using Chromquest V 2.53 software (Thermoquest). HIP (200 mg/L) and 5OH-HIP (50 mg/L), supplied by MSD Oss, The Netherlands, were used as authentic samples.

### Macrophage infection assays

The human monocyte cell line U937 [66] was grown in RPMI 1640 (Invitrogen) + NaHCO3 (1 g/L) + sodium pyruvate (0.11 g/L) + glucose medium (4.5 g/L) (RPMI 1640 medium), buffered with 10 mM HEPES (Hopax fine chemicals, Taiwan) and supplemented with penicillin (200 IU/ml), streptomycin (200 IU/ml) and 10% fetal bovine serum (FBS). The cells were grown in suspension at 37°C and 5% CO2. For the macrophage survival assay, monocytes were grown for several days as described above. The culture medium was replaced with fresh culture medium and the cells were activated overnight with phorbol 12-myristate 13-acetate (60 ng/ml, PMA, Sigma-Aldrich) to induce their differentiation to macrophages. The differentiated cells were spun down (5 min at 200 x g) and the pellet was re-suspended in fresh, antibiotic free RPMI 1640 medium with 10% FBS. For each strain to be tested, a tube containing 10 ml of a cell suspension (approximately 10^6^ cells/ml) was inoculated with *R. equi*, pre-grown in nutrient broth (Difco, Detroit, MI., USA) at 37°C, at a multiplicity of infection (MOI) of approximately 10 bacteria per macrophage. The bacteria were incubated with the macrophages for 1 h at 37°C and 5% CO2. The medium was replaced with 10 ml RPMI1640 medium supplemented with 10% FBS and 100 µg/ml gentamycin and incubated again for 1 h to kill any extra-cellular bacteria. In assays with complemented mutant strains of RE1Δ*ipdAB* and RE1Δ*fadE30* ampicillin (100 µg/ml) was added in addition to gentamycin (100 µg/ml), since the apramycin cassette conferred gentamycin resistance. The minimal inhibitory concentration (MIC) for ampicillin was determined at 1.5–2 µg/ml ampicillin for wild type and all mutant and complemented mutant strains using an ampicillin Etest strip (AB Biodisk/bioMérieux, Solna, Sweden). The macrophages (with internalized *R. equi*) were spun down (5 min at 200 x g) and the pellet was re-suspended in 40 ml RPMI1640 medium, buffered with 10 mM HEPES and supplemented with 10% FBS and 10 µg/ml gentamycin, plus 10 µg/ml ampicillin in assays with the complemented mutant strains. This suspension was divided over four culture bottles (10 ml each) and incubated at 37°C and 5% CO2. After 4, 28, 52 and 76 h the macrophages (one culture bottle per strain per time point) were spun down (5 min at 200 x g) and the pellet washed twice in 1 ml antibiotic free RPMI1640 medium. Finally the pellet was lysed with 1% Triton X-100 (Sigma-Aldrich) in 0.01M phosphate buffered saline, followed by live count determination (plate counting).

### Intratracheal challenge of foals with *R. equi* RE1Δ*ipdAB*

Six 3 to 5-week-old foals were allotted with mare to two groups of three foals, ensuring an even distribution of age over the groups. At T = 0 all foals were challenged with 100 ml suspension of RE1Δ*ipdAB* or *R. equi* RE1 (control) by trans-tracheal injection. Bacterial suspensions of *R. equi* strains RE1 or RE1Δ*ipdAB* were made by plating onto blood agar (Biotrading Benelux, Mijdrecht, The Netherlands) and incubation for 24 h at 37°C. Bacteria were then harvested with 4 ml of sterile isotonic PBS per plate and diluted with sterile isotonic PBS to a final concentration of approximately 5×10^4^ CFU/ml. Live count determination by plate counting was performed post-challenge. Infectivity titers were determined at 4.3×10^4^ CFU/ml for RE1 and 7.1×10^4^ CFU/ml for RE1Δ*ipdAB*. Foals were examined daily post-challenge until necropsy for clinical signs using a numerical clinical scoring system with 13 parameters (Table S3). The clinical score was calculated as the sum of clinical scores of the 13 different parameters. At day 21 post-challenge a post-mortem examination was performed. The foals were euthanized by anaesthesia with xylazine (100 mg/100 kg) and ketamine (500 mg/100 kg) and subsequent bleeding to death. The lungs were weighed in order to calculate the lung to body weight ratio. Details of these examinations are described below for the immunization experiment.

### Oral immunization of foals and subsequent challenge with virulent strain *R. equi* 85F

Oral immunization of foals was based on a study done by Hooper McGrevy *et al*. (2005) [53] with modifications. Eight 2 to 4-week-old foals were allotted to two groups of four foals each, ensuring an even distribution of age over the groups. During the experiment the foals suckled and the mares were fed according to standard procedures. *R. equi* strain RE1Δ*ipdAB* was administered orally (1 ml) to the foals for vaccination at T = 0 and a booster at T = 14 days. The infectivity titer of RE1Δ*ipdAB* was determined by plate counting (8.7×10^7^ CFU/ml and 4.1×10^7^ CFU/ml for the first and second vaccination, respectively). *R. equi* strain 85F (CNCM I-3250; [52], [64]) was used as challenge strain and plated onto blood agar and incubated for 24 h at 37°C. Bacteria were harvested with 4 ml of sterile isotonic PBS per plate and diluted with sterile isotonic PBS to a final concentration of approximately 5×10^4^ CFU/ml. At T = 28 days all foals were challenged with 100 ml *R. equi* 85F by trans-tracheal injection. Live count determination by plate counting was performed post-challenge in order to confirm the infectivity titer. Foals were examined daily for clinical signs using the numerical clinical scoring system described above (Table S3). Foals were weighed and blood was sampled at day of vaccination, day of challenge and at day of necropsy. Serum antibody titers against *R. equi* were determined as follows. *R. equi* strain 85F cell wall extract was prepared by resuspension of cells in 2% Triton X-114. The detergent phase containing VapA and other surface molecules (13.5 mg protein/ml) was diluted 2000x in 40 mM PBS and coated to microtiter plates during 16 h at 37°C. After washing with 40 mM PBS + 0.05% Tween, serial dilutions of test sera were made in the wells. After incubation for 1 h at 37°C and subsequent washing, the bound antibodies were quantified using HRP-rec protein G conjugate and 3,3′,5,5′-tetramethylbenzidine (TMB) as substrate. The antibody titers in sera were calculated using a positive standard serum with a defined titer of 9 (log_2_) as reference. Rectal swabs for bacterial re-isolation were sampled just before each vaccination and on frequent days after vaccination. The swab samples were serially diluted in physiological salt solution and plated on blood agar and incubated at 37°C for 16–24 h. *R. equi* colonies were initially identified by the typical non-hemolytic mucoid colony morphology, enumerated and expressed as CFU/ml.

At day 14 (controls; T = 42 days) or day 17–20 (vaccinates; T = 45–48 days) post-challenge foals were euthanized. The lungs were weighed in order to calculate the lung to body weight ratio. A complete post-mortem examination was performed with special attention to the lungs and gut with associated lymph nodes. Tissue samples (1 cm^3^) were excised from seven standard sites representative of the lobes of each half of the lung (3 sites per half and the accessory lobe); diseased tissue was preferentially selected for each site. The mirror image samples (the two samples of the equivalent lobe on each half) were pooled to give three samples per foal and a sample of the accessory lobe. Each (pooled) sample was homogenized, serially diluted and inoculated on blood agar plates and then incubated at 37°C for 16–24 h. *R. equi* colonies were enumerated and expressed as CFU/ml homogenate.

### Ethics statement

This study was carried out in strict accordance with the recommendations of the “Dutch Experiments on Animal Act”. The protocol was approved by the Committee on the Ethics of Animal Experiments of Intervet International bv (Permit Number: REV 07060).

### Statistical analysis

The *R. equi* counts (log_10_ CFU/ml) after incubation with macrophages for 4, 28, 52 and 76 h, reflecting the survival rate, were statistically analysed by ANOVA using a linear mixed model for repeated measurements and including time zero counts as covariate in the model Verbeke and Molenberghs [67]. Advanced statistical methods were applied for the ordinal scores over time of the daily clinical score using Generalized Estimating Equations (GEE with p-values based on empirical standard error) and ANOVA for repeated measurements for continuous outcomes of rectal temperature, lung scores (% consolidation) and the quantitative re-isolation of *R. equi* from the different lung lobes. In these methods the correlation of the repeated measurements on subjects (i.e. animals) is taken into account. Statistical methods were conducted in SAS V9.1 (SAS Institute Cary, NC, USA) using two-sided tests and a significance level (α) of 0.05.

## Supporting Information

Supplemental Figure S1 Schematic overview of the cholesterol catabolic gene cluster in *Rhodococcus equi* 103S and *Mycobacterium tuberculosis* H37Rv [36]. Grey arrows represent reciprocal homologues. White arrows represent genes for which no reciprocal homologue is present. Black arrows indicate the *ipdA*, *ipdB*, *fadE30* and *fadA6* genes that were studied by mutational analysis.
(TIF)

Supplemental Table S1 Sequence identities of *Rhodococcus equi* 103S and *Mycobacterium tuberculosis* H37Rv proteins encoded by the cholesterol catabolic gene cluster.
(DOC)

Supplemental Table S2 Oligonucleotides used in this study.
(DOC)

Supplemental Table S3 Numerical clinical scoring system using 13 parameters. Footnote a: for raw data collection record measured value. Footnote b: record joint(s) with effusion (synovitis) e.g. hock, fetlock, carpi.
(DOC)
