# Supplementary material for: The Steroid Catabolic Pathway of the Intracellular Pathogen Rhodococcus equi Is Important for Pathogenesis and a Target for Vaccine Development
Source: PLoS Pathog. 2011 Aug 25;7(8):e1002181. doi: 10.1371/journal.ppat.1002181 (PMC3161971; doi:10.1371/journal.ppat.1002181)
Supplement: Supplemental Table S1 — Sequence identities of Rhodococcus equi 103S and Mycobacterium tuberculosis H37Rv proteins encoded by the cholesterol catabolic gene cluster. (DOC) [file ppat.1002181.s002.doc]

**Supplemental table 1.** Sequence identities of *Rhodococcus equi* 103S and *Mycobacterium tuberculosis* H37Rv proteins encoded by the cholesterol catabolic gene cluster.

| *R. equi* 103S  Protein | *M. tuberculosis* H37Rv Best hit | Gene name | Amino acid identity (%) |
| --- | --- | --- | --- |
| REQ_06490 | Rv3574 | *kstR* | 65 |
| REQ_06500 | Rv3573 | *fadE34* | 56 |
| REQ_06520 | Rv3544 | *fadE28* | 50 |
| REQ_06530 | Rv3543 | *fadE29* | 71 |
| REQ_06540 | Rv3542 | *-* | 62 |
| REQ_06550 | Rv3541 | *-* | 69 |
| REQ_06560 | Rv3540 | *ltp2* | 78 |
|  |  |  |  |
| REQ_06760 | Rv3538 | *hsd4B* | 59 |
| REQ_06770 | Rv3537 | *kstD* | 65 |
| REQ_06780 | Rv3527 | *-* | 28 |
| REQ_06790 | Rv3526 | *kshA* | 64 |
| REQ_06800 | Rv3570 | *hsaA* | 75 |
| REQ_06810 | Rv3569 | *hsaD* | 70 |
| REQ_06820 | Rv3568 | *hsaC* | 75 |
| REQ_06830 | Rv3567 | *hsaB* | 71 |
|  |  |  |  |
| REQ_06980 | Rv3564 | *fadE33* | 44 |
| REQ_06990 | Rv3563 | *fadE32* | 49 |
| REQ_07000 | Rv3562 | *fadE31* | 63 |
| REQ_07010 | Rv1935 | *echA13* | 41 |
| REQ_07020 | Rv3561 | *fadD3* | 52 |
| REQ_07030 | Rv3560 | *fadE30* | 65 |
| REQ_07040 | Rv3559 | *-* | 71 |
| REQ_07050 | Rv3557 | *kstR2* | 59 |
| REQ_07060 | Rv3556 | *fadA6* | 71 |
|  |  |  |  |
| REQ_07150 | Rv3553 | *-* | 68 |
| REQ_07160 | Rv3552 | *ipdB* | 67 |
| REQ_07170 | Rv3551 | *ipdA* | 69 |
| REQ_07180 | Rv3550 | *echA20* | 72 |
| REQ_07190 | Rv3549 | *-* | 58 |
| REQ_07200 | Rv3548 | *-* | 70 |
| REQ_07210 | Rv0730 | *-* | 44 |
| REQ_07220 | Rv3547 | *-* | 55 |
| REQ_07230 | Rv3546 | *fadA5* | 74 |
| REQ_07240 | Rv3545 | *cyp125* | 62 |
| REQ_07250 | Rv0760 | *-* | 32 |
| REQ_07260 | Rv0940 | *-* | 40 |
| REQ_07270 | Rv3523 | *ltp3* | 75 |
| REQ_07280 | Rv3522 | *ltp4* | 70 |
| REQ_07290 | Rv3521 | *-* | 59 |
| REQ_07300 | Rv3520 | *-* | 69 |
| REQ_07310 | Rv1144 | *-* | 75 |
| REQ_07320 | Rv3556 | *-* | 47 |
| REQ_07330 | - | *-* | - |
| REQ_07340 | Rv3516 | *echA19* | 69 |
| REQ_07350 | Rv3515 | *fadD19* | 65 |
| REQ_07360 | Rv1533 | *-* | 48 |
| REQ_07370 | Rv3506 | *fadD17* | 51 |
| REQ_07380 | Rv3505 | *fadE27* | 55 |
| REQ_07390 | Rv3504 | *fadE26* | 76 |
| REQ_07410 | Rv3503 | *fdxD* | 55 |
| REQ_07420 | Rv3502 | *hsd4A* | 59 |
| REQ_07430 | Rv3501 | *supA* | 64 |
| REQ_07440 | Rv3500 | *supB* | 65 |
| REQ_07450 | Rv3499 (*mce4A*) | *mce2A* | 40 |
| REQ_07460 | Rv3498 (*mce4B*) | *mce2B* | 46 |
| REQ_07470 | Rv3497 (*mce4C*) | *mce2C* | 39 |
| REQ_07480 | Rv3496 (*mce4D*) | *mce2D* | 38 |
| REQ_07490 | Rv3495 (*mce4E*) | *mce2E* | 37 |
| REQ_07500 | Rv3494 (*mce4F*) | *mce2F* | 40 |
| REQ_07510 | Rv3493 (*mce4H*) | *mas2A* | 28 |
| REQ_07520 | Rv3492 (*mce4I*) | *mas2B* | 26 |
