# Supplementary material for: The Steroid Catabolic Pathway of the Intracellular Pathogen Rhodococcus equi Is Important for Pathogenesis and a Target for Vaccine Development
Source: PLoS Pathog. 2011 Aug 25;7(8):e1002181. doi: 10.1371/journal.ppat.1002181 (PMC3161971; doi:10.1371/journal.ppat.1002181)
Supplement: Supplemental Table S2 — Oligonucleotides used in this study. (DOC) [file ppat.1002181.s003.doc]

**Supplemental Table S2.** Oligonucleotides used in this study.

| **PCR** | **PCR Amplicon** | **Size (bp)** | **Oligonucleotide sequence (nr.)** |
| --- | --- | --- | --- |
| 1 | Upstream region *R. equi* RE1 *ipdAB*  (construction pSelAct-ipd1) | 1,368 | ipdABequiUP-F TGCCGCTGACGGAGGAGATCAT  ipdABequiUP-R GATATCATACCGGCGACTGCCTCATCCA |
| 2 | Downstream region *R. equi* RE1 *ipdAB*  (construction pSelAct-ipd1) | 1,396 | ipdABequiDOWN-F GATATCGAACCACCCGTGGTCACCAAC  ipdABequiDOWN-R TCGAGCAGCGAACTGGCCTGAA |
| 3 | Upstream region *R. equi* RE1 *ipdAB*  (confirmation Δ*ipdAB* mutant) | 1,726  (wt: 3,067) | ipdABequiContrUP-F AGTCCGACGACGATCGAGTTGA  ipdABequiContr-R TCACGCCGAGACCTCACGGTCA |
| 4 | Downstream region *R. equi* RE1 *ipdAB*  (confirmation Δ*ipdAB* mutant) | 1,682  (wt: 3,023) | ipdABequiContr-F ATGGCTGAGAAGCGCGACAAGC  ipdABequiContrDOWN-R TCGTCGTCGTCTCGCACCAGAT |
| 5 | *ipdAB* genes *R. equi* RE1  (confirmation Δ*ipdAB* mutant) | 296  (wt: 1,636) | ipdABequiContr-F ATGGCTGAGAAGCGCGACAAGC  ipdABequiContr-R TCACGCCGAGACCTCACGGTCA |
| 6 | *ipd* operon including *echA20* and *ipdAB*  (complementation of Δ*ipdAB* mutant) | 4,453 | ipdABequiContrUP-F AGTCCGACGACGATCGAGTTGA  ipdABequiContrDOWN-R TCGTCGTCGTCTCGCACCAGAT |
| 7 | Upstream region *R. equi* RE1 *ipdA2B2*  (construction pSelAct-ΔipdAB2) | 1,444 | ipdAB2equiUP-F TCGAGGTGGTTCATGACGAAGA  ipdAB2equiUP-R AGATCTCCGGCCGACCACCTCTTTCTCC |
| 8 | Downstream region *R. equi* RE1 *ipdA2B2*  (construction pSelAct-ΔipdAB2) | 1,387 | ipdAB2equiDOWN-F AGATCTAGTGCGGAGGAGCTGGAACTGA  ipdAB2equiDOWN-R ACTAGTGATCTCGTCCGTGACCTGATG |
| 9 | Upstream region *R. equi* RE1 *ipdA2B2*  (confirmation Δ*ipdA2B2* mutant) | 1,557  (wt: 3,053) | ipdAB2ContUP-F CCGACATCACGGTGTCGGGATC  ipdAB2Contr-R TCACGCGGGAACCTCCTTGTCG |
| 10 | Downstream region *R. equi* RE1 *ipdA2B2*  (confirmation Δ*ipdA2B2* mutant) | 1,471  (wt: 2,967) | ipdAB2Contr-F TTGTCGGACAAGAGAATGTCGG  ipdAB2ContDOWN-R GGTCGTGACGTCCGCGGTGTTC |
| 11 | *ipdA2B2* operon *R. equi* RE1  (confirmation Δ*ipdA2B2* mutant) | 123  (wt: 1,619) | ipdAB2contr-F TTGTCGGACAAGAGAATGTCGG  ipdAB2contr-R TCACGCGGGAACCTCCTTGTCG |
| 12 | Upstream region *R. equi* RE1 *fadE30*  (construction pSelAct-fadE30) | 1,511 | fadE30equiUP-F TCCATTCGCGCCAGCGCATTCT  fadE30equiUP-R AGATCTCTTCGAGCCATTCGCGAAT |
| 13 | Downstream region *R. equi* RE1 *fadE30*  (construction pSelAct-fadE30) | 1,449 | fadE30equiDOWN-F AGATCTACGGCGGATCCAACGAGAT  fadE30equiDOWN-R AGGTCGCGGAACTCCTGGTTAC |
| 14 | Upstream region *R. equi* RE1 *fadE30*  (confirmation Δ*fadE30* mutant) | 1,866  (wt: 2,909) | fadE30UPcontr-F ACGATGTACGCACGACCGACCT  fadE30contr-R GACAGCTTCTCGACGGTCTCAC |
| 15 | Downstream region *R. equi* RE1 *fadE30*  (confirmation Δ*fadE30* mutant) | 1,767  (wt: 2,811) | fadE30contr-F AGGCGAGGCGGAACCTCTATAC  fadE30DOWNcontr-R CGTCCAGAACGATGGAGAGGTA |
| 16 | *fadE30* locus *R. equi* RE1  (confirmation Δ*fadE30* mutant) | 428  (wt: 1,470) | fadE30contr-F AGGCGAGGCGGAACCTCTATAC  fadE30contr-R GACAGCTTCTCGACGGTCTCAC |
| 17 | *fadE30* full-length gene  (complementation of Δ*fadE30* mutant) | 2,837 | fadE30equiUP-F TCCATTCGCGCCAGCGCATTCT  fadE30contr-R GACAGCTTCTCGACGGTCTCAC |
| 18 | Upstream region *R. equi* RE1 *fadA6*  (construction pSelAct-fadA6) | 1,429 | fadA6equiUP-F AGTCGTTCGTCTGCGACGTCTC  fadA6equiUP-R agatctGGCCACCGTTCTTCTTGCCGAT |
| 19 | Downstream region *R. equi* RE1 *fadA6*  (construction pSelAct-fadA6) | 1,311 | fadA6equiDOWN-F agatctGTTACGGCCTGCTCACCATCTG  fadA6equiDOWN-R GTGCACGCGATGCTCGAATTCC |
| 20 | Upstream region *R. equi* RE1 *fadA6*  (confirmation Δ*fadA6* mutant) | 1,570  (wt: 2,584) | fadA6UPcontr-F TCTCGGTGAGACCGTCGAGAAG  fadA6contr-R GAGGACTGGTCGGACTACTTCT |
| 21 | Downstream region *R. equi* RE1 *fadA6*  (confirmation Δ*fadA6* mutant) | 1,705  (wt: 2,719) | fadA6contr-F TGGCCAAGCAGTACCTCTCCAT  fadA6DOWNcontr-R CTGTTCACTCGGCTGCAGAAGG |
| 22 | *fadA6* locus *R. equi* RE1  (confirmation Δ*fadA6* mutant) | 236  (wt: 1,250) | fadA6contr-F TGGCCAAGCAGTACCTCTCCAT  fadA6contr-R GAGGACTGGTCGGACTACTTCT |
| 23 | *vapA R. equi* RE1  (confirmation presence virulence plasmid) | 408 | vapA-F GCAGCAGTGCGATTCTCAATAG  vapA-R TAACTCCACCGGACTGGATATG |
