# Supplementary material for: The Steroid Catabolic Pathway of the Intracellular Pathogen Rhodococcus equi Is Important for Pathogenesis and a Target for Vaccine Development
Source: PLoS Pathog. 2011 Aug 25;7(8):e1002181. doi: 10.1371/journal.ppat.1002181 (PMC3161971; doi:10.1371/journal.ppat.1002181)
Supplement: Supplemental Table S3 — Numerical clinical scoring system using 13 parameters. Footnote a: for raw data collection record measured value. Footnote b: record joint(s) with effusion (synovitis) e.g. hock, fetlock, carpi. (DOC) [file ppat.1002181.s004.doc]

**Supplemental Table S3** Numerical clinical scoring system using 13 parameters.

|  |  |  |  |
| --- | --- | --- | --- |
| General impression | 0 = normal | Respiration rate^a^ | 0 = ≤ 70 |
|  | 1 = slightly depressed |  | 1 = 71-80 |
|  | 2 = moderately depressed |  | 2 = 81-90 |
|  | 3 = severely depressed |  | 3 = 91-100 |
|  |  |  | 4 = >100 |
| Anorexia | 0 = normal |  |  |
|  | 4 = loss of appetite | Respiration type | 0 = costoabdominal |
|  |  |  | 2 = slightly abdominal |
| Temperature^a^ | 0 = ≤ 38.5 |  | 4 = abdominal |
|  | 1 = 38.6-39.0 |  | 6= strongly abdominal |
|  | 2 = 39.1-39.5 |  |  |
|  | 3 = 39.6-40.0 | Palpation larynx | 0 = no coughing |
|  | 4 = 40.1-40.5 |  | 1 = one or two coughs |
|  | 5 = 40.6-41.0 |  | 2 = several coughs |
|  | 6 = >41.0 |  |  |
|  |  | Palpation trachea | 0 = no coughing |
| Nasal discharge | 0 = absent |  | 1 = one or two coughs |
|  | 1 = serous |  | 2 = several coughs |
|  | 2 = mucopurulent |  |  |
|  | 3 = purulent | Spontaneous cough | 2 = after inspection |
|  | (if excessive 1 point extra) |  | 4 = before inspection |
|  |  |  |  |
| Ocular discharge | 0 = absent | Auscultation | 0 = normal |
|  | 1 = serous |  | 2 = slight rales |
|  | 2 = mucopurulent |  | 4 = dry or wet rales |
|  | 3 = purulent |  | 6 = areas with no sound |
|  | (if excessive 1 point extra) |  |  |
|  |  | Effusion of joints^b^ | 0 = normal |
| Heart beat / min^a^ | 0 = ≤ 90 |  | 1 = mild |
|  | 1 = 91-100 |  | 2 = moderate |
|  | 2 = 101-110 |  | 3 = severe |
|  | 3 = 111-120 |  |  |
|  | 4 = >120 | Other | Record abnormality |

^a^ for raw data collection record measured value

^b^ record joint(s) with effusion (synovitis) e.g. hock, fetlock, carpi
